# Synergistic impact of mesoporous Zn/Al-LDH nanorods for developing dielectric and thermal properties of epoxy as insulation in GIS/GIL

**DOI:** 10.1038/s41598-025-34294-8

**Published:** 2026-01-27

**Authors:** M. Ramadan, Mahmoud Ezzat, Mousa. A. Abd-Allah, S. M. A. El-Gamal, Abdelrahman Said

**Affiliations:** 1 Chemistry Department, Faculty of Science, Ain Shams University, Cairo, 11566 Egypt; 2 Department of Electrical Engineering, Faculty of Engineering at Shoubra, Benha University, Cairo, 11672 Egypt; 3 Faculty of Computer Science, Benha National University (BNU), Al Obour, Egypt

**Keywords:** Layered double hydroxide, Nanoparticles, Epoxy resin, Dielectric reliability, Breakdown strength, Chemistry, Engineering, Materials science, Nanoscience and technology

## Abstract

Gas-insulated switchgear (GIS) and transmission lines (GIL) depend on epoxy spacers to ensure electrical reliability under high-voltage stress. Yet, the limited dielectric strength and thermal tolerance of neat epoxy constrain its long-term applicability. This work investigates Zn/Al layered double hydroxide (LDH) nanoparticles as functional fillers for epoxy insulation. The nanofillers were synthesized by co-precipitation, surface-functionalized for improved compatibility, and incorporated into epoxy at loadings of 1–7 wt%. Structural, thermal, dielectric, and breakdown properties were systematically assessed. The Zn/Al-LDH exhibited a mesoporous nanostructure with high surface area (~ 98.9 m^2^/g) and uniform dispersion in the matrix, increasing epoxy structural ordering from 21.1% to 28.9%. Thermal stability improved as the char yield rose from 14% (neat epoxy) to 17.1% at 5 wt%, while the glass transition temperature shifted from 93.5 °C to 109.8 °C, surpassing the IEC 62271-1 limit. Dielectric analysis confirmed stable permittivity, low loss at 50 Hz, and suppressed DC conductivity down to 10⁻^15^–10⁻^12^ S/cm. Most notably, breakdown strength rose from 30.08 kV/mm in neat epoxy to 37.42 kV/mm at 5 wt%, representing a 24% enhancement and the most reliable statistical profile under Weibull analysis. Beyond this concentration, agglomeration effects reversed the benefits, lowering performance below that of pure epoxy. These findings highlight Zn/Al-LDH/epoxy nanocomposites as a promising class of advanced insulating materials, with optimum performance achieved at 3–5 wt% loading. The combined improvements in dielectric reliability, thermal stability, and breakdown endurance demonstrate their potential for next-generation GIS/GIL applications.

## Introduction

Gas-Insulated Switchgear (GIS) and Gas-Insulated Transmission Lines (GIL) are essential components of modern high-voltage power networks. Their importance arises from their compact design, high operational reliability, and safe performance in space-constrained environments. The increasing use of GIS/GIL in urban substations and critical grid nodes reflects the need for materials that provide high dielectric strength while keeping the equipment footprint small^1^. In these systems, sulfur hexafluoride (SF_6_) or SF_6_-based gas mixtures serve as the primary insulating medium. In contrast, epoxy spacers play a dual role: they hold the conductors in position and manage the local gas–solid dielectric interface, which strongly influences field distribution and insulation reliability under electrical stress^2^.

Epoxy has long been the material of choice for spacers in GIS/GIL because it combines electrical insulation strength, mechanical robustness, chemical stability, and the ability to be moulded into complex geometries. Moreover, epoxy bonds well with metallic inserts, which are essential for maintaining the electro-mechanical stability of spacer assemblies. To further improve its performance, researchers have investigated epoxy nanocomposites. The addition of carefully selected inorganic nanofillers can adjust interfacial polarization and trap energy levels, suppress conductivity growth, and enhance resistance to electrical ageing provided that filler type, loading, and dispersion are well controlled^2–4^.

Initial efforts using micro-scale fillers such as SiO₂ and Al₂O₃ primarily targeted improvements in mechanical strength and mitigation of thermal expansion mismatch in epoxy composites. However, these systems delivered only limited gains in electrical performance. The emergence of nanotechnology has since enabled transformative advances. Nanofillers including SiO₂, Al₂O₃, TiO₂, and ZrO₂ exhibit strong interfacial interactions even at low concentrations, which can significantly influence dielectric permittivity (ε′), loss tangent (tan δ), trap distribution, and breakdown strength^2^.

The electrical performance of nanofilled epoxy systems is highly sensitive to filler composition and loading. For instance, epoxy/SiO₂ composites at 3–5 wt% exhibited a decline in DC breakdown strength, while epoxy/MgO systems at similar concentrations showed marked improvement. Epoxy/Al₂O₃ nanocomposites also demonstrated enhanced breakdown behavior, attributed to the formation of well-dispersed particle networks that suppress charge accumulation and disrupt discharge pathways^3^. These findings highlight the importance of optimizing both filler morphology and concentration, as excessive loading or poor dispersion can lead to agglomeration and void formation ultimately compromising electrical reliability.

The dielectric properties of epoxy strongly govern its suitability as an insulating matrix, and numerous reports have explored how nanofillers alter the relative permittivity (ε′), dielectric loss (tan δ), and electrical conductivity (σ). Neat epoxy typically exhibits a very low conductivity in the order of 10^−16^ to 10^−15^ S m⁻^1^ and a high relative permittivity at low frequencies such as 50 Hz^4^. When high-permittivity ceramics are introduced, substantial improvements are observed. For example, epoxy/BaTiO₃ composites at 0.5 volume fraction displayed ε′ values exceeding 40, compared with values below 10 for the neat resin. However, this increase is coupled with higher losses; tan δ values may rise above 0.1 at high filler contents^5^.

In contrast, oxide nanofillers such as SiO₂ often suppress ε′, tan δ, and σ at modest loadings^2,3,6^. For instance, epoxy with 5 wt% SiO₂ exhibited lower permittivity and loss than the neat system, whereas increasing to 10 wt% reversed this trend and led to higher values^7^. These contrasting behaviors underscore the importance of filler selection and optimization in tailoring epoxy-based insulation systems for specific electrical performance requirements.

Frequency-dependent studies further reveal characteristic relaxation signatures, with tan δ minima near 10^2^ Hz and growth toward the MHz range. Similar interfacial immobilization effects are reported for Al₂O₃/epoxy, where ε′ decreases at ~ 1 wt% due to rigid interfacial layers, rises at 2–4 wt%, and then falls again beyond 5 wt%^8^. These trends underscore the dual role of nanofillers: high-permittivity ceramics can significantly elevate ε′, while oxide-based fillers tend to suppress dielectric loss and conductivity by restricting interfacial polarization. Nevertheless, the overall dielectric response remains highly sensitive to filler type, loading concentration, and interfacial compatibility, necessitating careful optimization for targeted insulation performance.

Among oxide nanofillers, systems based on SiO₂, TiO₂, and Al₂O₃ have shown improved breakdown strength and reduced dielectric loss when particle size and surface chemistry are optimized. Surface functionalization (for example, with silane coupling agents like APTES) further enhances performance by improving filler dispersion and introducing chemical groups that act as carrier traps. Particle size is also critical; studies on TiO₂-based nanocomposites showed that smaller nanoparticles provided higher breakdown strength compared with larger particles, highlighting the influence of interfacial area and defect statistics^6^.

Thermal characterization of epoxy nanocomposites reveals that neat epoxy typically undergoes initial degradation between 300–350 °C, with major weight loss occurring from 350–450 °C. The incorporation of nanofillers can effectively delay thermal degradation onset and increase char yield, although inadequate dispersion may negate these benefits. Differential scanning calorimetry (DSC) places the glass transition temperature (Tg) of neat epoxy within the range of 72–99 °C. Numerous studies have shown that nanofillers such as SiO₂, Al₂O₃, TiO₂, and LDH not only elevate Tg but also reduce the heat capacity change (ΔCp), indicating restricted polymer chain mobility. Overall, thermal enhancements are evident but remain highly dependent on filler type, concentration, and dispersion quality^6^.

Layered double hydroxide (LDH) nanofillers have garnered significant attention due to their unique lamellar morphology and tunable chemical composition. These features enable precise modulation of trap states within epoxy matrices, positioning LDH as a promising additive for suppressing surface charge accumulation and enhancing dielectric reliability in GIS spacer applications^9^.

Recent studies have established LDH nanofillers as multifunctional additives capable of improving both the dielectric and thermal behavior of epoxy systems. Huan et al.^10^ have demonstrated that silane-modified LDH particles suppress electrode charge injection and reduce field distortion by optimizing trap energy distributions, thereby enhancing insulation reliability. Similarly, Liu et al.^11^ have integrated Mg/Al-LDH-coated silica spheres into epoxy coatings, achieving superior thermal insulation and flame retardancy, confirming the multifunctional potential of LDH structures. Moreover, integrated experimental and simulation evidence has demonstrated that Zn/Al-LDH nanofillers effectively suppress surface charge accumulation and enhance flashover voltage in GIS spacers, linking material-level improvements with system-level insulation reliability^12^. Despite these advancements, studies exploring the mesoporous Zn/Al-LDH nanorods architecture in epoxy matrices remain scarce, particularly concerning its combined influence on dielectric reliability, thermal stability, and breakdown strength which forms the central focus of the present work.

Therefore, this study aims to develop high-performance epoxy-based insulating materials for GIS and GIL applications by incorporating mesoporous Zn/Al layered double hydroxide (LDH) nanorods. The work focuses on enhancing overall insulation reliability, combining superior dielectric and thermal performance with improved breakdown strength, thereby addressing the inherent limitations of neat epoxy under high-voltage stress and advancing the design of next-generation insulation systems.

## Materials

The base epoxy used in this work is KEMAPOXY 150 JM (Chemicals for Modern Building International, Giza, Egypt), a two-component, solvent-free liquid epoxy formulated for electrical insulation. It complies with ASTM D150, ASTM D257, and ASTM D495 standards, with a density of 1.15 ± 0.02 kg/L at 25 °C. It consists of two Components, the epoxy A and the hardener B, which were mixed in a 3:1 weight ratio, providing a pot life of ~ 45 min, initial setting within 10–12 h, and complete curing after 7 days. The specifications of all the chemicals used in this study are listed in Table 1.

**Table 1:** Chemicals specifications used in this study.

Material	IUPAC name	Purity (%)	Producer (City, Country)
Epoxy resin	Bisphenol-A diglycidyl ether (DGEBA)	–	Chemicals for Modern Building International, Giza, Egypt
Hardener	Polyamine curing agent	–	Chemicals for Modern Building International, Giza, Egypt
Zinc nitrate	Zinc nitrate hexahydrate	98%	Sigma-Aldrich, St. Louis, USA
Aluminum chloride	Aluminum chloride	99.999%	Sigma-Aldrich, St. Louis, USA
Sodium carbonate	Sodium carbonate	≥ 99.5%	Sigma-Aldrich, St. Louis, USA
Silane coupling agent	(3-Aminopropyl) triethoxysilane (APTES)	99%	Sigma-Aldrich, St. Louis, USA
Ethanol	Ethanol	99%	El Nasr Pharmaceutical Chemicals, Cairo, Egypt

### Preparation of Zn/Al-LDH nanofillers

**Fig. 1:**
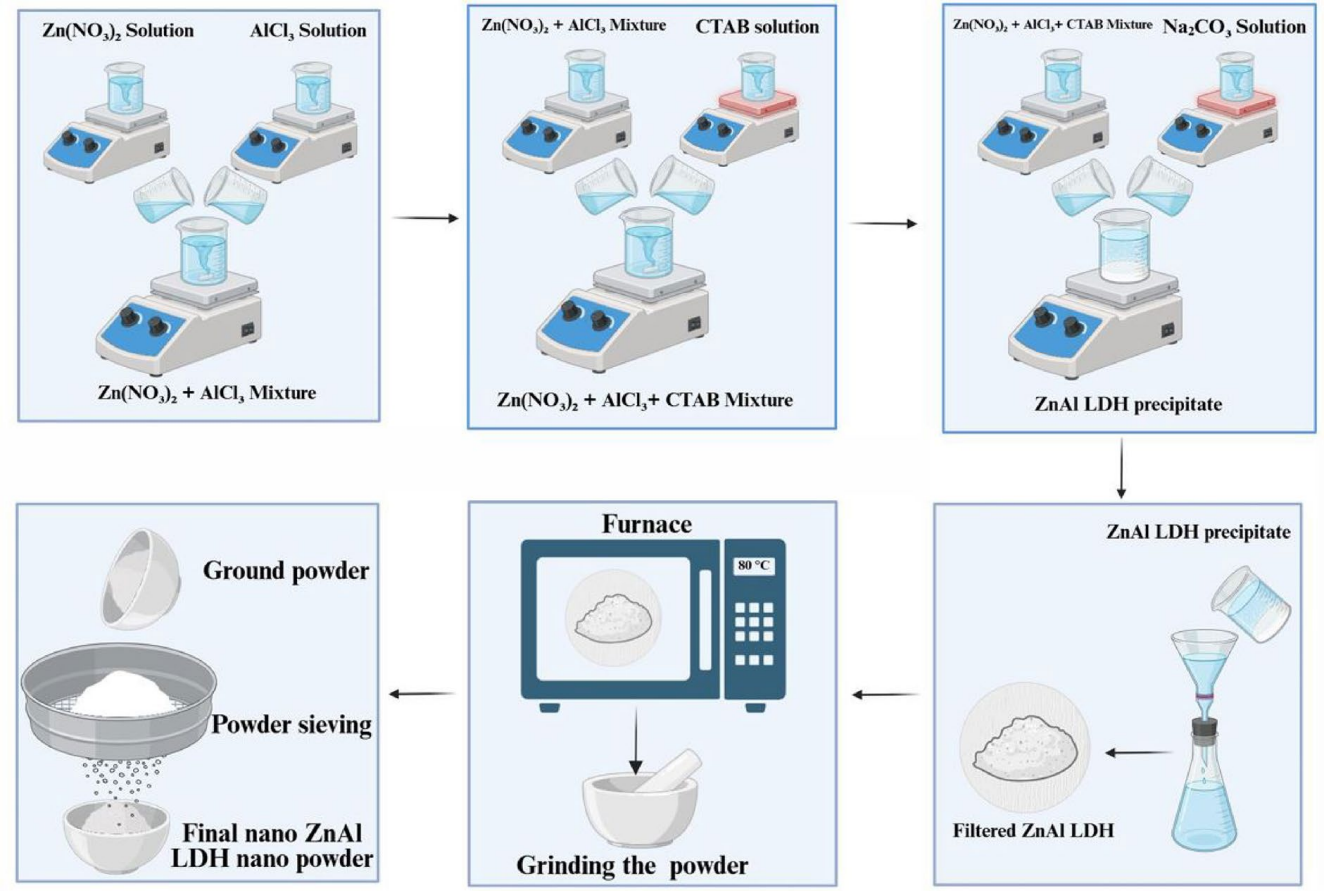
A full description of the nano material preparation process.

Zn–Al layered double hydroxide (Zn–Al–CO₃ LDH) was synthesized by the co-precipitation method. Equimolar solutions of zinc nitrate [Zn(NO₃)₂, 0.5 M] and anhydrous aluminium chloride [AlCl₃, 0.5 M] were prepared in a 2:1 molar ratio and stirred magnetically for 15 min to ensure homogeneity. A 2 M sodium carbonate solution was then added dropwise to the mixture until a white precipitate formed at pH 7–8. The obtained precipitate was filtered, thoroughly washed with distilled water, and dried overnight at 80 °C. Finally, the dried product was ground and sieved to obtain Zn–Al–CO₃ LDH powder. A full description of the Zn/Al-LDH preparation process is illustrated in Fig. 1.

### Surface functionalization of nanoparticles

**Fig. 2:**
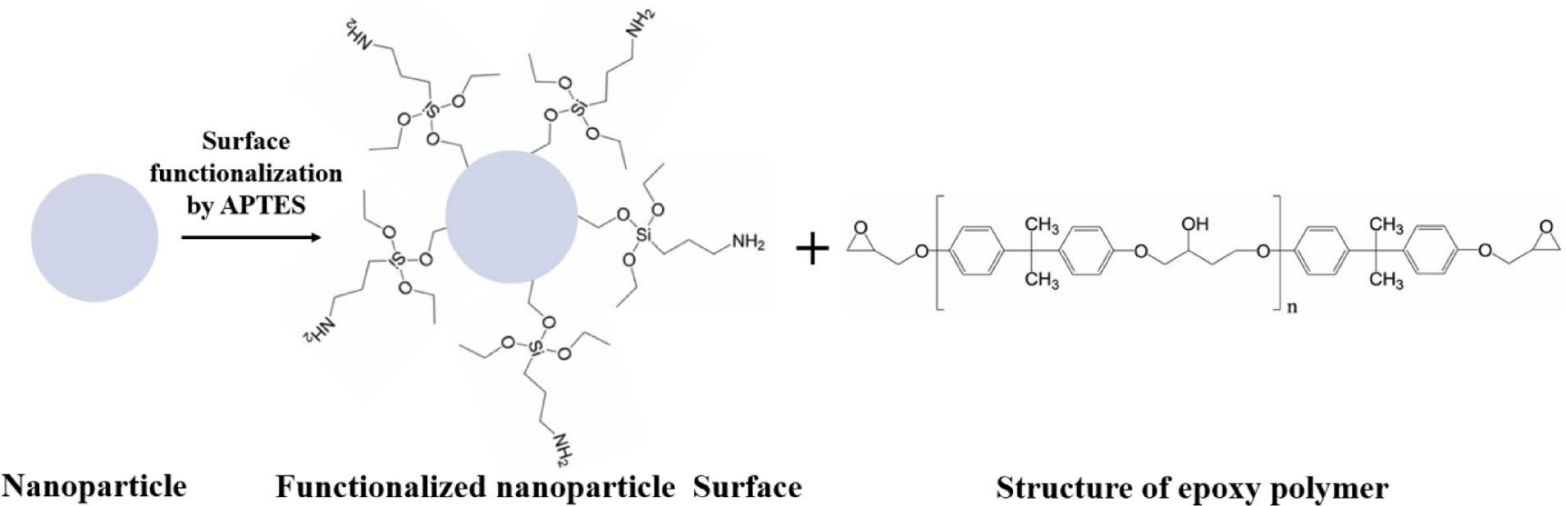
Surface functionalization process.

Surface functionalization of nanoparticles is an effective strategy to enhance their dispersibility in epoxy matrices by reducing surface energy and improving compatibility with the polymer. Unlike physical treatments, which are unstable under heat or solvents, chemical modification using coupling agents such as silane (APTES) provides stable and durable interfaces, as shown in Fig. 2. These agents form bonds both with the nanoparticle surface and the polymer chains, reducing hydrophilicity and promoting uniform dispersion. As a result, functionalization improves interfacial adhesion and tailors the bulk properties of nanocomposites^6^.

The functionalization process of the nanoparticles was initiated by dispersing 3 g of the prepared powder in 60 mL of ethanol within a round-bottom flask. The suspension underwent ultrasonication at 70 °C for 30 min to disintegrate agglomerates and achieve uniform dispersion, followed by mechanical stirring at 500 rpm for 30 min under the same conditions. A 10% (w/w) solution of 3-aminopropyltriethoxysilane APTES in ethanol was subsequently added dropwise while stirring continued, and the reaction mixture was maintained for 5h to promote effective grafting of silane molecules onto the nanoparticle surfaces. After completion, the product was recovered by filtration, washed thoroughly with ethanol to eliminate unreacted silane, and dried at 80 °C overnight, resulting in silane-modified nanoparticles suitable for incorporation into the epoxy matrix^13^.

### Preparation of epoxy Zn/Al-LDH nanocomposite

**Fig. 3:**
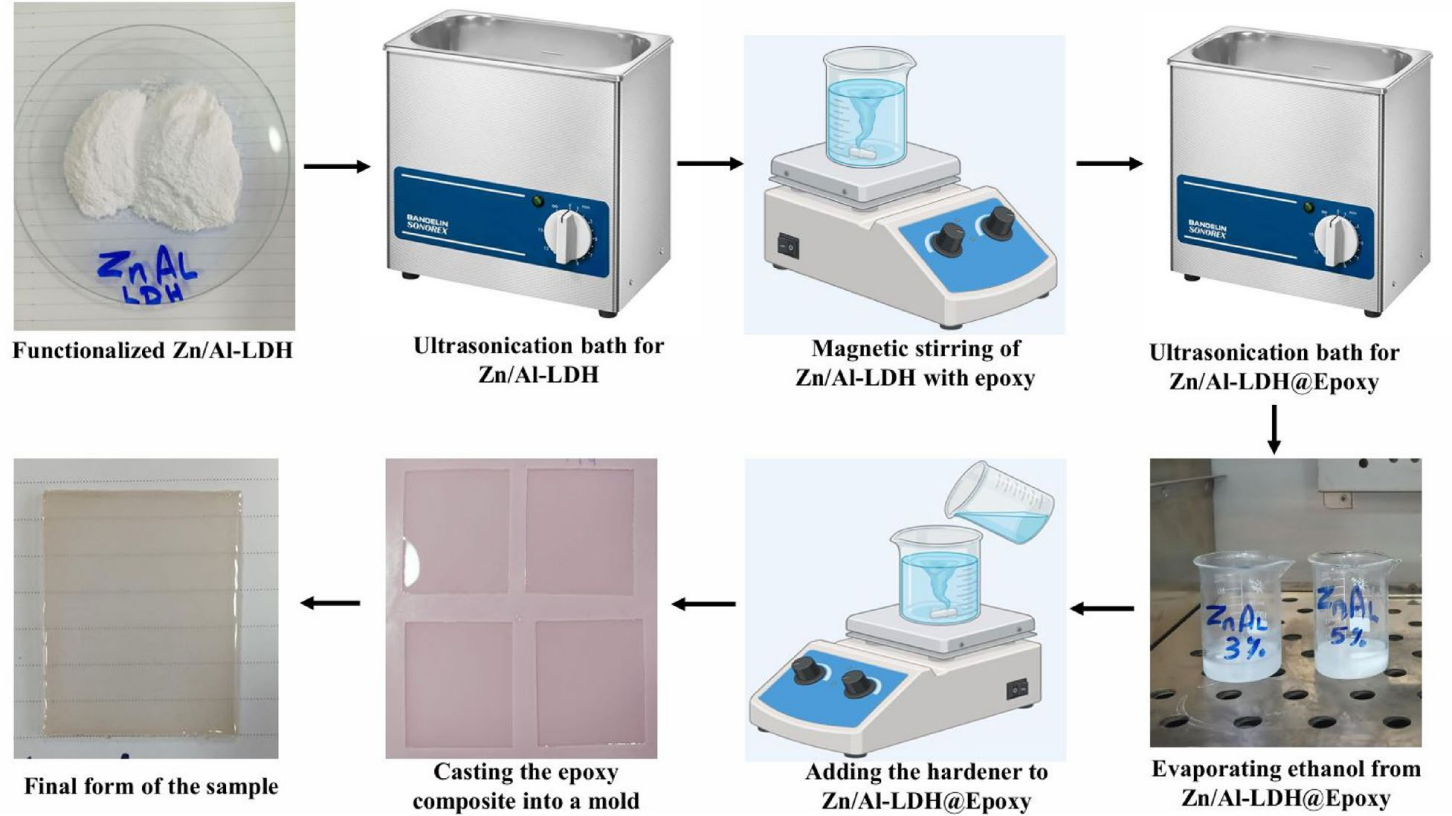
A schematic illustration of the key preparation stages.

The epoxy-based nanocomposites were prepared through a multi-step procedure. Initially, the synthesized Zn/Al–LDH nanoparticles were dried at 80 °C overnight to remove residual moisture, then dispersed in ethanol and ultrasonicated for 60 min to achieve uniform suspension. The obtained dispersion was gradually introduced into epoxy resin under continuous stirring for 2 h, followed by an additional ultrasonication step (30 min) and mechanical stirring (2 h) to improve homogeneity. Residual ethanol was eliminated by heating the mixture at 80 °C until constant weight was achieved. The solvent-free epoxy/nanoparticle mixture was then blended with the hardener at a 3:1 weight ratio, stirred for 3 min, and degassed on a hot plate to remove entrapped air bubbles. Finally, the mixture was cast into pre-prepared molds and cured under ambient conditions for 24 h to obtain the nanocomposite samples. A schematic illustration of the key preparation stages is presented in Fig. 3.

## Methodology

After the preparation of Zn/Al–LDH nanoparticles and epoxy resin nanocomposites at different loadings (1, 3, 5, and 7%wt.), a series of characterization and performance evaluations were carried out for both nanomaterials and nanocomposites.

### Nanoparticle characterization

The morphology of Zn/Al–LDH nanoparticles was examined using a High Resolution Transmission Electron Microscope coupled with Selected Area Electron Diffraction (HRTEM/SAED, Model 2100 Plus, JEOL Ltd., Tokyo, Japan) operated at 200 kV to determine particle size, dispersion state and crystallographic structure, The degree of crystallinity and phase composition were confirmed by X-ray Diffraction (XRD, Model X’Pert PRO, PANalytical B.V., Almelo, Netherlands) using Cu Kα radiation (λ = 1.5406 Å), scanned in the 2θ range of 5–70° with a step size of 0.02° equipped with Cu Kα radiation (λ = 1.5406 Å), operated at 40 kV and 30 mA. The scans were recorded in Bragg–Brentano geometry over a 2θ range of 5–70° with a step size of 0.02° and scan rate of 2°/min. In addition, all textural parameters of synthesized nanoparticles were evaluated via nitrogen adsorption desorption technique. Surface area and porosity measurements were performed using a Micromeritics ASAP 2020 Plus Accelerated Surface Area and Porosimetry System (Micromeritics Instrument Corp., Norcross, GA, USA).

### Nanocomposite characterization

The microstructure of the epoxy Zn/Al–LDH nanocomposites was observed by Scanning Electron Microscopy equipped with Energy Dispersive X-ray Spectroscopy using (SEM, Model Quanta 250, FEI Company, Hillsboro, USA, equipped with EDX, Oxford Instruments, Abingdon, UK) to assess nanoparticle dispersion and elemental composition. Complementary XRD measurements were also carried out on the composites to investigate any structural modifications upon filler incorporation.

### Thermal analysis

The thermal behavior of the optimized nanocomposite formulation (corresponding to the loading that exhibited the highest breakdown strength) was evaluated using Thermogravimetric Analysis (TGA/DSC Analyzer, THEMSYS ONE, Setaram Instrumentation, Caluire-et-Cuire, France). Tests were conducted from room temperature up to 700 °C at a heating rate of 15 °C/min under nitrogen flow. TGA was used to study thermal stability and degradation profiles, while DSC provided the glass transition temperature (T_g_) of the composites. The second heating run was used to eliminate any residual solvent or absorbed moisture effects. The recorded heat flow values were normalized to the polymer mass fraction.

### Dielectric properties

Dielectric measurements, including relative permittivity (ε_r_), dielectric loss tangent (tan δ), and AC conductivity, were performed using (Dielectric Spectrometer, Alpha-A Analyzer with Concept 40 Interface, Novocontrol Technologies GmbH & Co. KG, Montabaur, Germany). Impedance and dielectric spectroscopy were performed using a parallel-plate electrode system integrated inside the Novocontrol sample cell, which is the standard configuration recommended for dielectric materials. The epoxy discs were placed between two stainless-steel parallel plate electrodes with direct mechanical contact. The cell is connected to the Alpha-A analyzer with DETACHEM software. This configuration is suitable for low-loss dielectric materials and is fully compliant with Novocontrol’s specifications for broadband dielectric analysis. All dielectric measurements were performed at room temperature (25–27 °C) under ambient laboratory atmosphere. Samples were placed inside the parallel-plate cell and allowed to equilibrate thermally for approximately 10 min before data acquisition then A constant AC excitation of 1 Vrms was applied to the samples over a frequency range of 10⁻^1^–10⁶ Hz.

### Breakdown strength

**Fig. 4:**
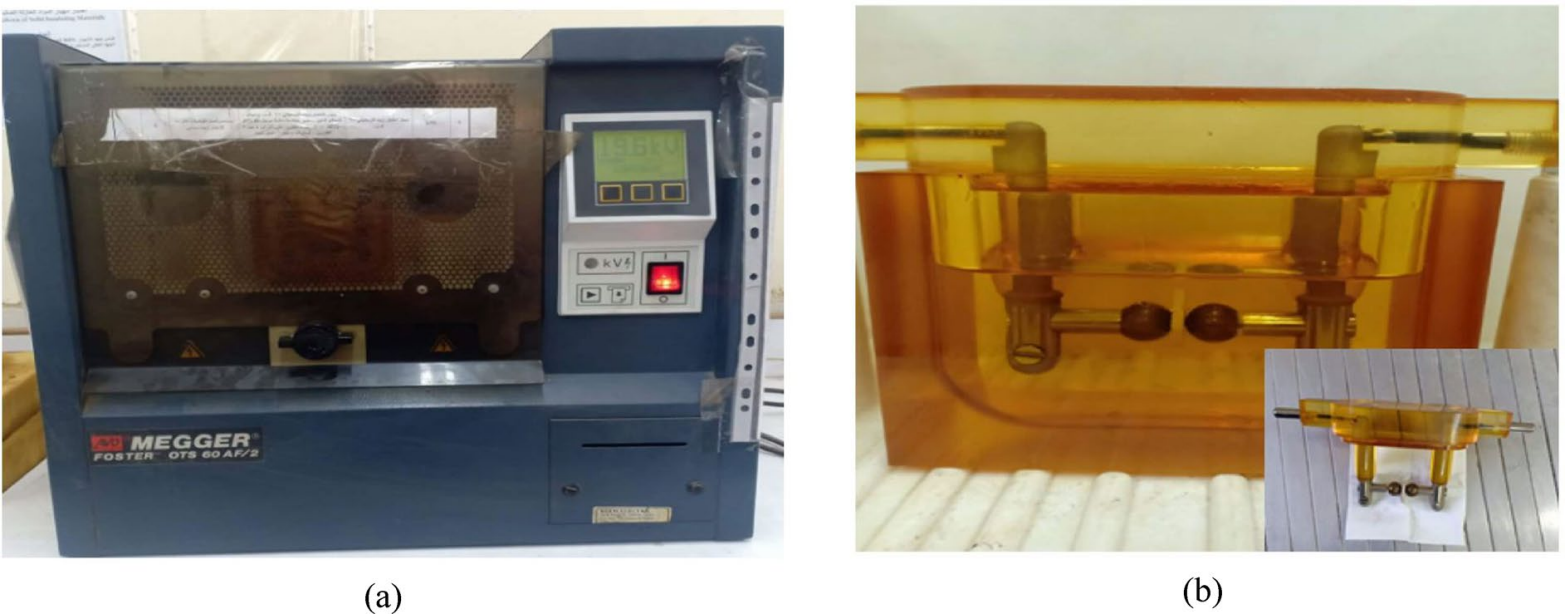
(**a**) Megger Foster 60AF/2 Oil tester, (**b**) Test step.

The AC breakdown strength of the epoxy Zn/Al–LDH nanocomposites was determined using (Breakdown Tester, Model Foster 60AF/2, Megger Ltd., Dover, UK) shown in Fig. 4. According to the procedure recommended by ASTM (D3755–14), a disk sample (5cm x 5cm) of 1 mm thickness was placed between two spherical copper electrodes and fully immersed in clean insulating oil within a standard oil-cell test fixture to suppress surface flashover. A 50 Hz sinusoidal voltage was applied and increased linearly at 500 V/s until dielectric failure occurred. Fifteen replicates per composition were tested to ensure statistical reliability. The breakdown data were analyzed using Weibull distribution to evaluate the reliability and dispersion of breakdown strength values across different filler loadings.

## Results and discussion

### Characterization of nanoparticles

To comprehensively elucidate the structural, morphological, and textural properties of the synthesized Zn/Al-layered double hydroxide (LDH) materials, a combination of advanced characterization techniques was employed, including X-ray diffraction (XRD), high-resolution transmission electron microscopy (HR-TEM) coupled with selected area electron diffraction (SAED), and nitrogen adsorption–desorption isotherm analysis.

**Fig. 5:**
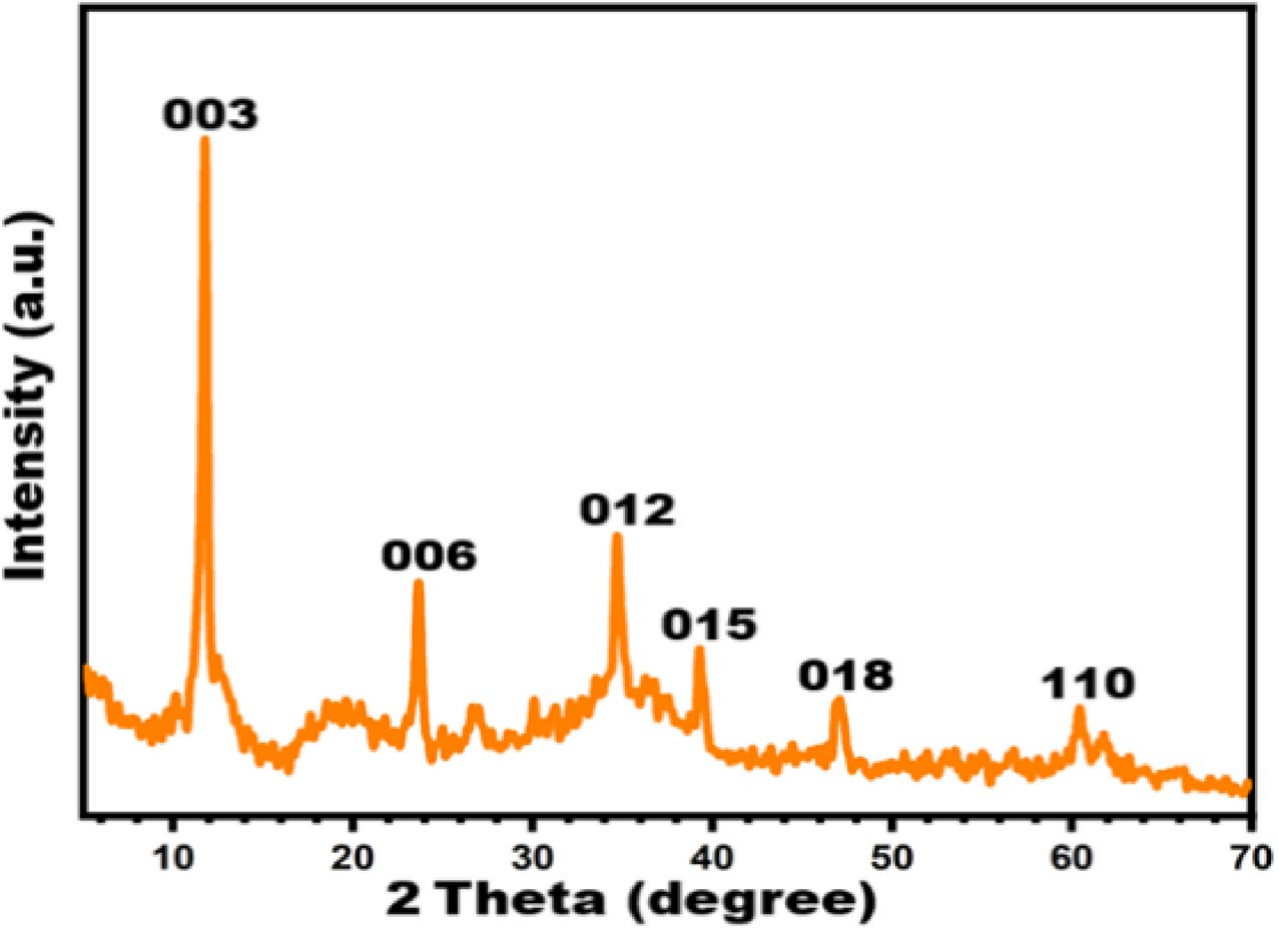
XRD patterns for synthesized Zn-Al-CO_3_ LDHs.

XRD analysis was employed to investigate the crystalline phase composition and structural characteristics of the synthesized Zn/Al-layered double hydroxide (LDH). As depicted in Fig. 5, the diffraction patterns clearly indicate the formation of two distinct hexagonal phases, identified as Zn_0.63_Al_0.37_(OH)_2_ (CO_3_)_0.185_·xH_2_O (PDF#00-048-1024) and Zn_0.61_Al_0.39_ (OH)_2_(CO_3_)_0.195_.x H_2_O (PDF#00-048-1025). These phases exhibit well-defined basal and non-basal reflections at 2θ values of 11.76° (003), 23.57° (006), 34.69° (012), 39.23° (015), 47.07° (018), and 60.43° (110), corresponding to interlayer spacings (d-values) of 7.53871 Å, 3.76327 Å, 2.58342 Å, 2.33959 Å, 1.93069 Å, and 1.52867 Å, respectively. These reflections confirm the presence of a well-ordered layered structure, characteristic of LDH materials.

**Fig. 6:**
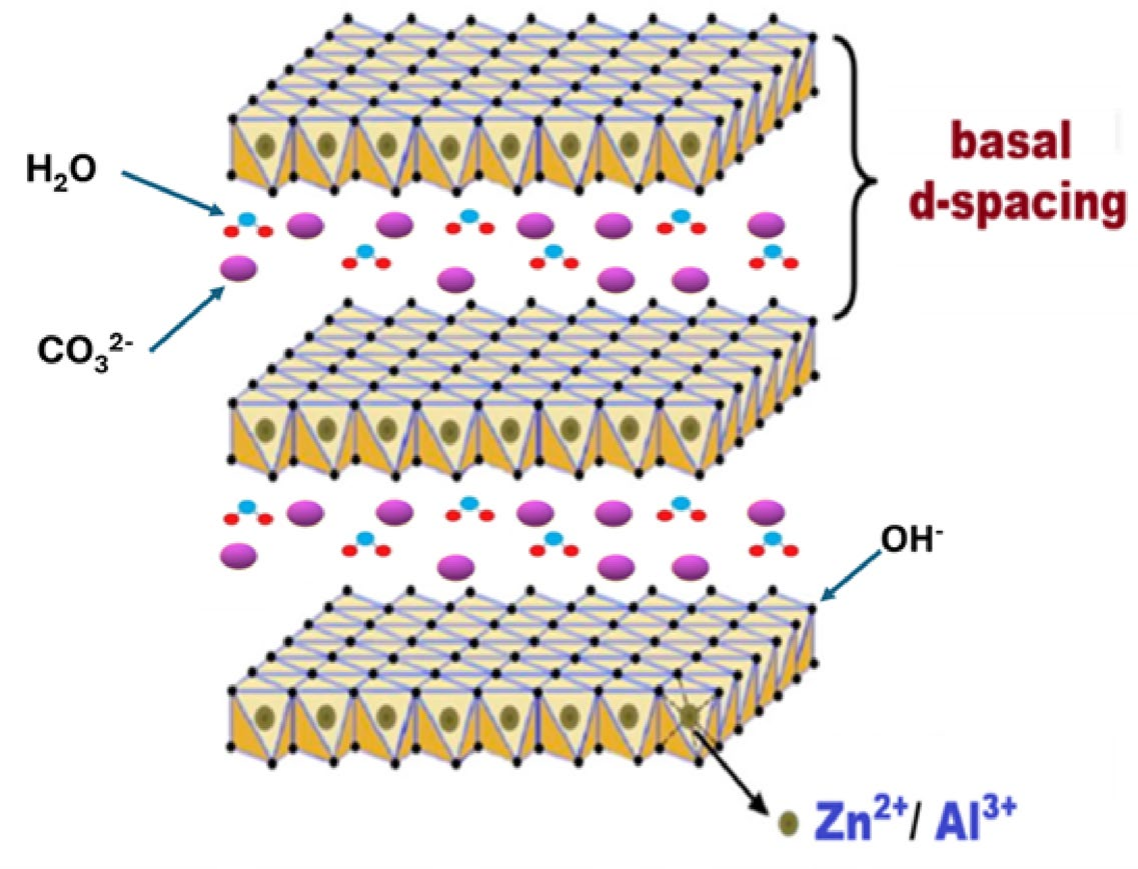
Lamellar architecture and brucite-like layers of synthesized Zn-Al-CO3 LDH.

Quantitative analysis using Match! software (version 3.15) determined an average crystallite size of approximately 33.7 nm, indicative of nanocrystalline domains. The calculated crystallinity degree of 50.65% suggests a semi-ordered structure, which is often advantageous for applications of electrical insulation. Structurally, the Zn-Al-CO₃ LDHs consist of positively charged brucite-like layers, ${[M}_{1-X}^{2+}$.${M}_{X}^{3+}$.(OH)_2_]X + , composed of Zn^2^⁺ and Al^3^⁺ cations, balanced by interlayer carbonate anions (CO₃^2^⁻) and water molecules^14–16^. This lamellar architecture (Fig. 6) inherently restricts the mobility of free charge carriers, thereby contributing to low electrical conductivity, a desirable trait for electrical insulation applications^17^. Furthermore, the low crystallinity and nanoscale crystallite size imply a high density of grain boundaries and structural defects, which act as electron scattering centers, further impeding charge transport^18^. The incorporation of Al^3^⁺ ions enhance the dielectric strength and thermal stability of the material, both of which are critical parameters for effective electrical insulation^19^.

High-resolution transmission electron microscopy (HR-TEM) analysis reveals that the synthesized Zn-Al-CO₃ layered double hydroxide (LDH) nanoparticles exhibit a predominantly fibrous and rod-like morphology, with entangled structures that are uniformly distributed across the observed field. The particles display well-defined edges and consistent dispersion, indicating a controlled synthesis process that effectively regulates particle growth. The measured dimensions of these nanostructures are consistently below 100 nm, confirming their nanoscale nature and suggesting a high surface-to-volume ratio. This morphological feature is particularly advantageous for insulation applications, where enhanced surface area contributes to improved thermal and dielectric properties^20^.

**Fig. 7:**
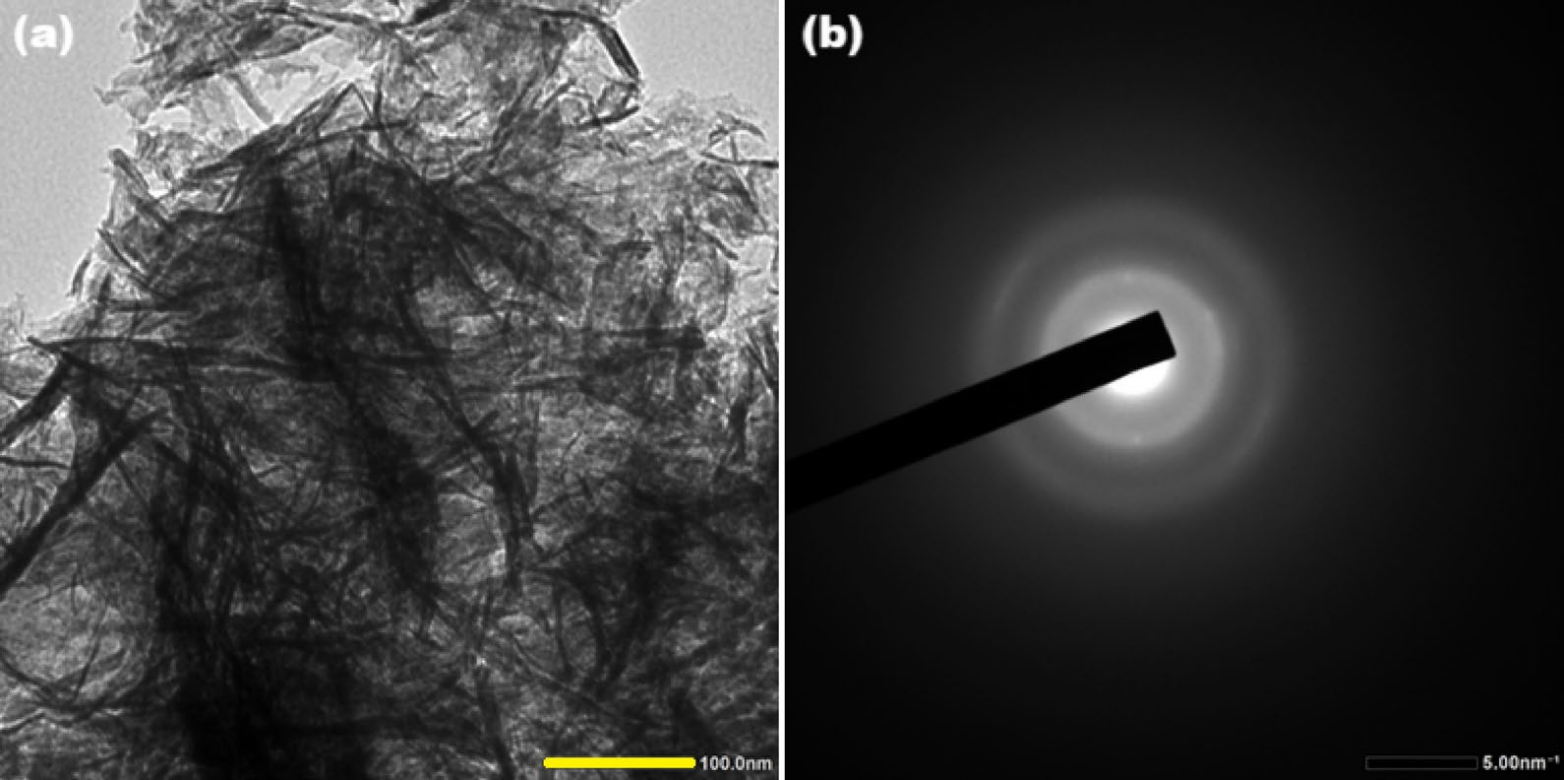
(**a**) HR-TEM and (**b**) SAED of synthesized Zn-Al-CO_3_ LDH.

To further assess the structural order of the nanoparticles, selected area electron diffraction (SAED) was employed. The resulting SAED patterns exhibit concentric diffraction rings, a hallmark of polycrystalline materials. These rings correspond to various crystallographic planes, confirming the presence of multiple crystalline domains within the LDH structure^21,22^. However, the relatively low intensity of the diffraction rings suggests that the material is not fully crystalline but rather exhibits a semi-crystalline nature. This intermediate crystallinity reflects a balance between ordered layer stacking and structural disorder; hence, the results obtained from HR-TEM/SAED techniques (Fig. 7) are in complete agreement with the XRD data.

**Fig. 8:**
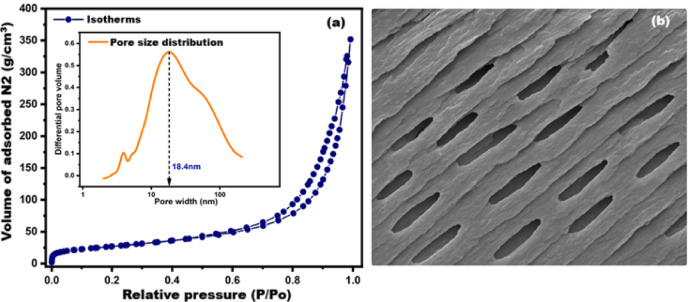
(**a**) Isotherms/pore size distribution of synthesized LDH and (**b**) a model for slit-like pores.

The nitrogen adsorption/desorption isotherms and pore size distribution of Zn/Al-CO₃ LDH, as depicted in Fig. 8, were systematically analyzed using the BET and BJH models to evaluate its textural characteristics^23,24^. The LDH exhibited a Type IV isotherm with an H3 hysteresis loop, in accordance with IUPAC classification, which is indicative of a mesoporous structure with slit-like pores typically associated with layered materials^25–28^.

This observation is fully consistent with the BET-derived parameters listed in Table 2, where the material demonstrates a notably high specific surface area of 98.893 m^2^ g⁻^1^ and a substantial total pore volume of 0.5279 cm^3^ g⁻^1^, indicating extensive accessible porosity.

**Table 2:** BET, and BJH characterization results.

Technique	Parameter	Value	Unit
BET	Specific surface area	98.893	m^2^ g⁻^1^
	Total pore volume	0.5279	cm^3^ g⁻^1^
BJH	Peak pore diameter	18.387	nm
	Average pore diameter	18.738	nm
	Median pore diameter	20.227	nm

The BJH pore-size distribution, presented in the inset of Fig. 8a, further supports the mesoporous nature of the material. As detailed in Table 2, the pore network is centered around a dominant peak at 18.387 nm, with an average pore diameter of 18.738 nm and a median pore diameter of 20.227 nm, collectively reflecting a narrow and unimodal mesopore distribution. Moreover, t-plot analysis confirms the absence of microporosity, reinforcing that the synthesized LDH is governed entirely by mesoporous texturing.

Importantly, Fig. 8b presents a schematic model for slit-like pores, which conceptually illustrates the expected pore geometry derived from the adsorption data.

These structural features are particularly advantageous for electrical insulation applications. The high surface area facilitates uniform dispersion of LDH particles within polymer matrices, enhancing interfacial adhesion and reducing the likelihood of agglomeration, which is critical for maintaining consistent dielectric properties^29^.

In addition, the mesoporous architecture may participate in charge-carrier moderation through increased interfacial area, although this effect likely acts in synergy with other mechanisms such as polarization at filler–matrix boundaries and improved structural ordering^30–32^.

The substantial pore volume also enables the incorporation of functional additives, such as flame retardants or conductive suppressors, which can be tailored to enhance the thermal stability and multifunctionality of the insulation system^33^. Overall, the textural characteristics of Zn/Al–CO₃ LDH contribute as part of a combined set of mechanisms that support dielectric enhancement and stability, rather than acting independently as the primary governing factor.

### Characterization of epoxy nanocomposites

To thoroughly investigate the phase composition, surface morphology, and thermal stability of the synthesized LDH@epoxy nanocomposite, a suite of advanced characterization techniques was systematically employed. These included X-ray diffraction (XRD) for identifying crystalline phases and structural integrity, scanning electron microscopy coupled with energy-dispersive X-ray spectroscopy (SEM/EDX) for detailed morphological and elemental analysis, and thermogravimetric analysis/differential scanning calorimetry (TGA/DSC) to assess thermal behavior, decomposition patterns, and heat flow characteristics. This multi-technique approach enabled a comprehensive understanding of the material’s structural and functional properties, which are critical for evaluating its performance in insulation applications.

**Fig. 9:**
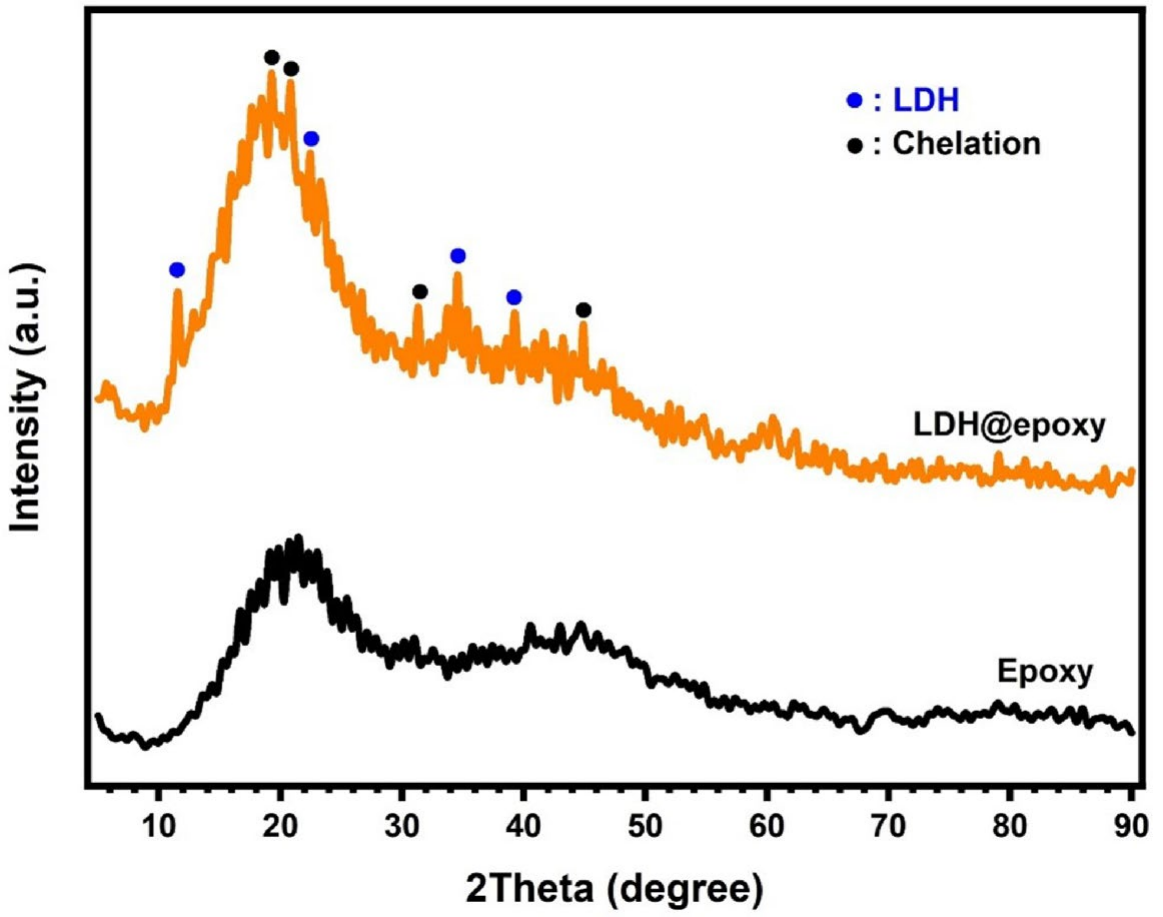
XRD patterns of pure epoxy and LDH@epoxy.

As illustrated in Fig. 9, the XRD analysis of pure epoxy (black curve) reveals two broad diffraction bands centered around 2θ ≈ 20° and 44°, characteristic of its predominantly amorphous nature. These broad features reflect the disordered molecular arrangement and lack of long-range crystalline order, which is typical of cross-linked thermosetting polymer systems^34^.

Upon incorporation of 5 wt% mesoporous Zn/Al-CO3-LDH nanorods into the epoxy matrix, the XRD pattern of the LDH@epoxy composite (orange curve) exhibits significant structural changes. Notably, new diffraction peaks emerge at 2θ ≈ 11.47°, 19.23°, 20.69°, 22.37°, 31.21°, 34.53°, 39.35°, and 44.31°, which are absent in the pure epoxy pattern. These peaks indicate the presence of crystalline LDH (PDF#00-048-1024) domains and suggest strong interfacial interactions between the LDH and epoxy matrix, likely due to polymer chain intercalation or chemical bonding that modifies the lattice spacing^35^.

The appearance of sharper and more intense peaks in the composite indicates an increase in structural ordering from 21.08% to 28.92%, as computed automatically through quantitative pattern analysis using Match! software (version 3.15).

Such modifications may also reflect internal stress or strain within the composite^36^, further confirming the successful integration of LDH. These structural enhancements are crucial for electrical insulation applications, as they contribute to improved dielectric behavior, reduced charge mobility through effective trapping, and greater thermal stability. Overall, the XRD results substantiate the reinforcing role of LDH in epoxy composites and underscore its potential for advanced insulation systems.

**Fig. 10:**
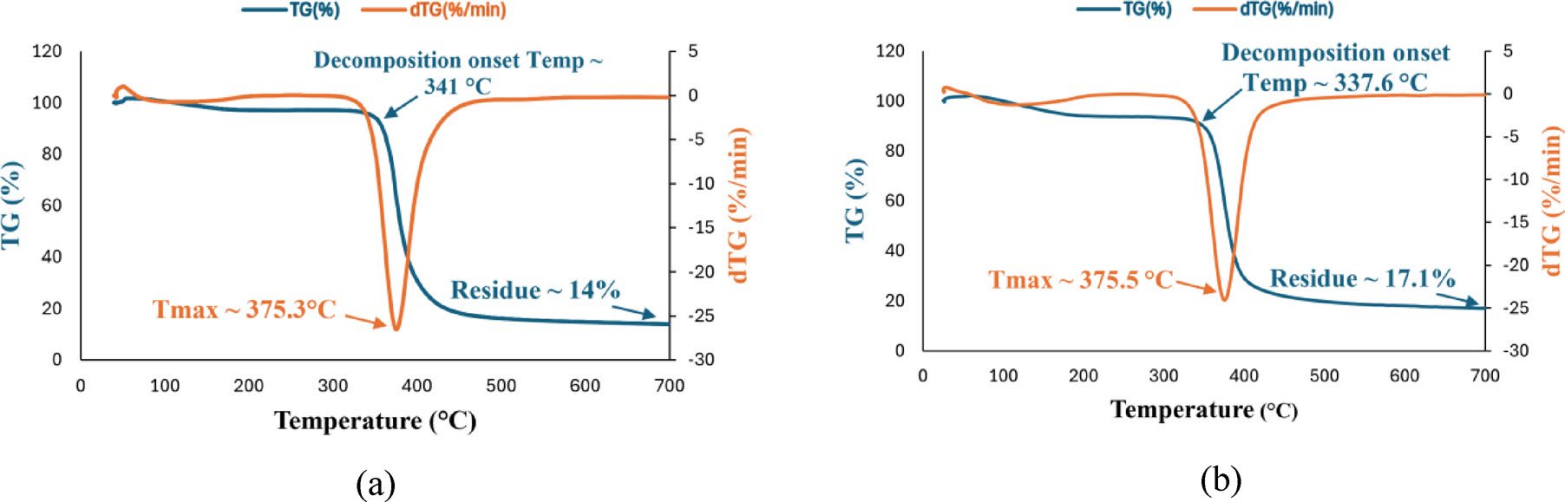
(**a**) TGA for pure epoxy sample, (**b**) TGA for epoxy Zn/Al-LDH (5%) nanocomposite.

Thermogravimetric analysis (TGA) and derivative thermogravimetry (DTG) profiles presented in Fig. 10a, b reveal distinct thermal behaviors between the neat epoxy and the epoxy nanocomposite containing 5 wt% Zn/Al-LDH. The neat epoxy (Fig. 10a) exhibits thermal stability up to approximately 300 °C, beyond which significant degradation initiates between 346–370 °C. The DTG curve shows a sharp peak at 375 °C, indicating the maximum rate of mass loss, with the primary decomposition occurring between 370–450 °C and leaving a residual char of ~ 14% at 700 °C.

In contrast, the LDH-modified epoxy (Fig. 10b) exhibits an early mass-loss event at ~ 172 °C, mainly associated with the release of physically adsorbed or interlayer water and a minor surface dehydroxylation of weakly bound hydroxyl groups. This behavior agrees with previous reports on LDH-containing systems, where an initial mass loss (50–250 °C) corresponds to dehydration and partial decomposition of hydroxide phases^15,16^. Despite this initial event, the main decomposition onset (~ 338 °C) and peak degradation temperature (375 °C) remain comparable to the neat epoxy. However, the final char residue increases to ~ 17.1%, signifying enhanced thermal stability. This improvement is consistent with the barrier effect and char-promoting properties of LDH nanofillers, which contribute to the formation of a more thermally resilient composite structure.

**Fig. 11:**
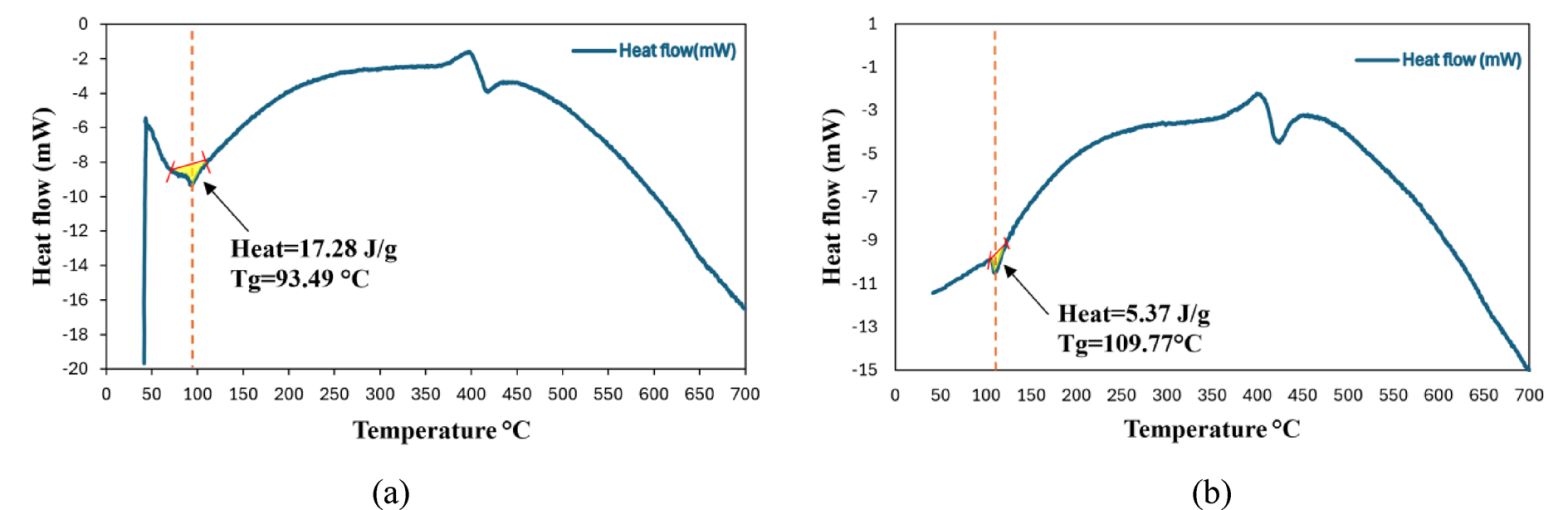
(**a**) DSC test for pure epoxy sample, (**b**) DSC test for epoxy Zn/Al-LDH (5%) nanocomposite.

Differential scanning calorimetry (DSC) is a standard thermal analysis for polymer characterization that records heat flow to/from a specimen. At the same time, its temperature is programmed under an inert or reactive purge. The technique resolves exothermic and endothermic events; the associated transition temperatures (e.g., glass transition, melting, evaporation, or decomposition). For amorphous polymers, the glass transition temperature (T_g_) marks the change from glassy to rubbery/viscous behavior and is accompanied by a heat-capacity step (ΔC_p_). In this work, the same DSC protocol will be applied to the neat epoxy and to the 5% Zn/Al-LDH/epoxy nanocomposite to compare Tg and ΔCp. Differential scanning calorimetry revealed a clear shift in the glass transition behavior upon the incorporation of Zn/Al-LDH nanofillers. For the neat epoxy in Fig. 11a, the glass transition temperature (Tg) was identified at 93.5 °C, accompanied by a relatively large enthalpic relaxation of approximately 17.3 J/g. This combination reflects the high mobility of polymer chain segments and the limited constraints within the pure epoxy network. In contrast, the nanocomposite containing 5 wt% Zn/Al-LDH in Fig. 11b exhibited a distinctly higher Tg of 109.8 °C, together with a markedly lower relaxation enthalpy of only 5.4 J/g. The upward shift in T_g_ indicates that the nanofiller restricts the segmental dynamics of the epoxy chains, consistent with enhanced interfacial interactions and increased effective crosslink density^37–39^.

At the same time, the pronounced reduction in ΔH implies that fewer cooperative chain motions occur during the transition, as the LDH platelets act as barriers to chain mobility and promote a more rigid network structure^37–39^. These findings corroborate the role of layered double hydroxides in improving the thermal stability and dimensional integrity of epoxy matrices under heating.

According to the IEC 62271-1 standard, the maximum allowable operating temperatures are 105 °C for the conductor that is in contact with the spacer material^40^. In this study, the neat epoxy exhibited a T_g_ of ~ 93.5 °C, which falls below this threshold. However, the incorporation of 5 wt% Zn/Al-LDH in epoxy successfully shifted T_g_ to ~ 109.8 °C, surpassing the IEC guideline.

**Fig. 12:**
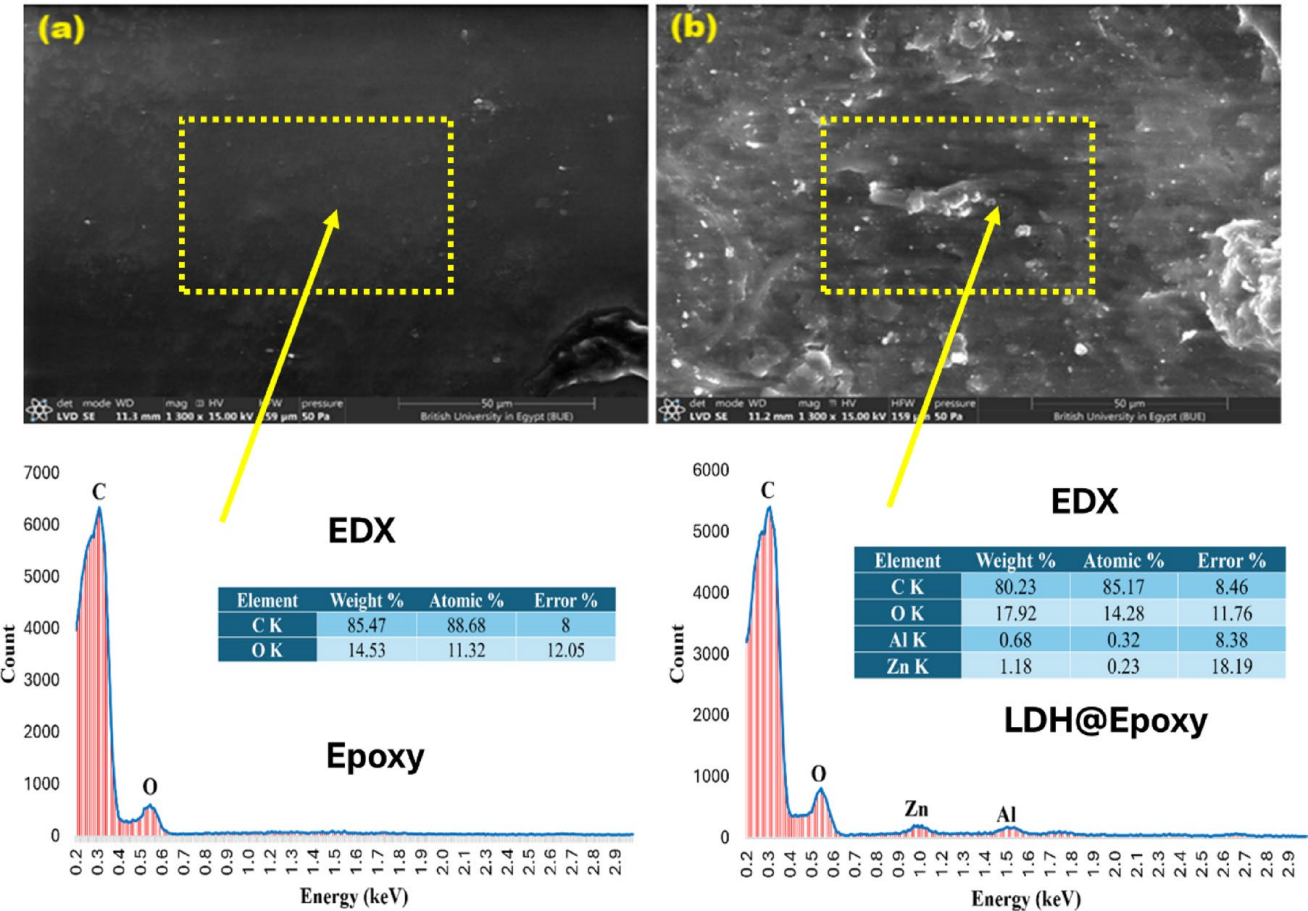
SEM/EDX images of (**a**) pure epoxy and (**b**) LDH@epoxy.

Figure 12 presents SEM/EDX micrographs of the neat epoxy (Fig. 12a) and LDH@epoxy (Fig. 12b). In the neat epoxy, the surface appears relatively smooth and homogeneous, with no distinct inorganic features or secondary phases. Only a few scattered bright spots can be observed, which are most likely attributed to preparation artefacts or surface debris rather than actual structural components. Overall, the micrograph confirms the purity of the epoxy matrix and provides a reliable baseline for comparison with the nanocomposite^41^. In contrast, the micrograph of LDH@epoxy reveals a markedly different surface morphology. Bright regions and irregularly shaped clusters are clearly distributed across the matrix, corresponding to the presence of LDH nanoparticles. Their enhanced contrast originates from the higher atomic number of Zn and Al compared to the carbon-rich epoxy background, resulting in stronger electron scattering. The occurrence of agglomerates is a common feature in polymer–nanofiller systems, arising from the high surface energy of nanoparticles. Nevertheless, the overall distribution of bright particles across the surface confirms the successful incorporation of the nanofiller into the epoxy. Energy-dispersive X-ray spectroscopy (EDX) was used to verify the elemental composition of the neat epoxy and the Epoxy/Zn–Al (LDH) composite, to check for contaminants, and to provide semi-quantitative weight/atomic percentages with instrument-reported errors^42^. The EDX spectrum for the neat epoxy shows only the polymeric constituents, with a dominant C Kα peak at ~ 0.28 keV and a weaker O Kα peak at ~ 0.53 keV; the semi-quantitative analysis gives C = 85.47 wt.% (88.68 at%) and O = 14.53 wt.% (11.32 at%), consistent with an organic matrix. After adding the Zn–Al LDH, two additional signals appear at the expected positions of Zn L (~ 1.01 keV) and Al Kα (~ 1.49 keV), confirming successful incorporation of the LDH-nanorod. The matrix still dominates, but the carbon fraction decreases to 80.23 wt%, accompanied by a rise in oxygen to 17.92 wt.%, which is consistent with the hydroxyl-rich LDH surface. Minor amounts of Zn = 1.18 wt.% (0.23 at%) and Al = 0.68 wt.% (0.32 at%) are detected; the larger uncertainty on Zn (18.19%) reflects its low concentration and the low-energy region’s higher absorption. No extraneous elements were observed above background, indicating a clean composite and validating the presence of Zn/Al-LDH within the epoxy.

The variations in C and O content between the neat epoxy and LDH@epoxy samples are fully consistent with the expected incorporation of the oxygen-rich LDH filler into the polymer matrix. The slight decrease in carbon (from 85.47 to 80.23 wt%) and corresponding increase in oxygen (from 14.53 to 17.92 wt%) reflect the presence of hydroxylated Zn–Al–LDH nanosheets, which contain abundant OH groups and metal oxygen bonds.

### Dielectric performance of epoxy nanocomposites

The subsequent section evaluates three essential dielectric characteristics of epoxy composites incorporating four varying concentrations of Zn/Al-LDH nanoparticles: relative permittivity, loss tangent (tan δ), and electrical conductivity.

#### Relative permittivity

**Fig. 13:**
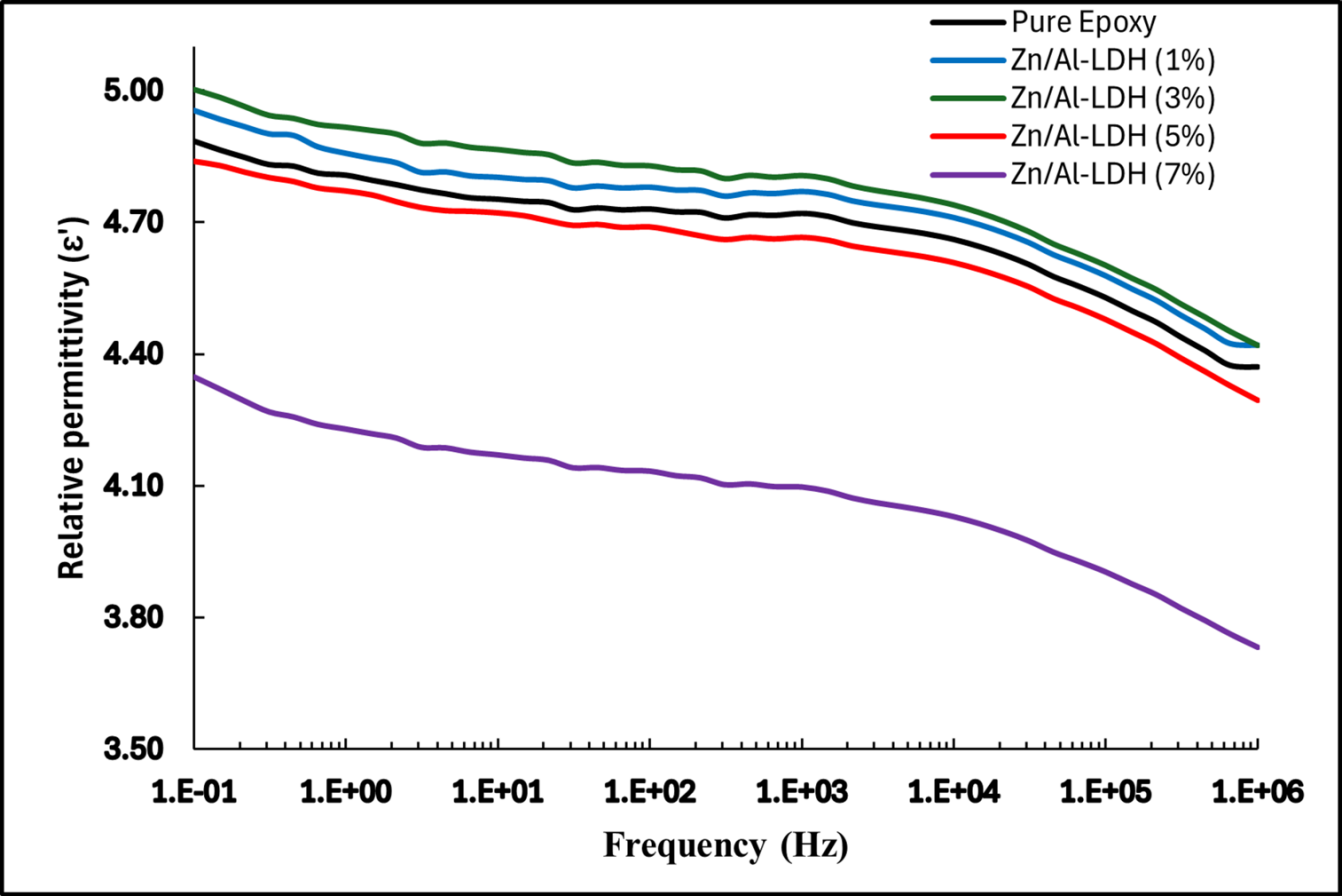
Variation of relative permittivity with frequency.

The frequency-dependent behavior of the relative permittivity (ε′) for epoxy nanocomposites filled with various weight percentages of Zn/Al-LDH exhibits a non-monotonic response at any frequency point, reflecting the complex interplay between interfacial polarization, filler dispersion, polymer chain dynamics, and nano-interfacial structure formation. Conversely, as shown in Fig. 13, for any single wt.% ε′ exhibits a typical monotonic decreasing trend with increasing frequency.

At low frequencies, the dipolar entities within the polymer matrix have sufficient time to orient themselves in response to the alternating electric field. This alignment with the changing field polarity leads to higher values of the relative permittivity. However, as the frequency increases, the dipoles can no longer follow the rapid field reversals, causing a gradual loss of polarization coherence and consequently a decrease in the relative permittivity with frequency.

However, a minor contribution from interfacial (Maxwell–Wagner–Sillars) polarization can still be expected at low frequencies, where charge accumulation at filler–matrix interfaces cannot be neglected. This interpretation is consistent with the model proposed by Xia et al. which demonstrated an inverse relationship between the interfacial (Maxwell–Wagner–Sillars) relative permittivity and frequency, arising from charge accumulation at the filler–matrix boundaries in heterogeneous polymer nanocomposites^43^.

The effect of filler concentration on ε′ reveals a nuanced dielectric response. At Low concentrations, 1 and 3 wt.% of Zn/Al-LDH, the permittivity slightly increased compared to the neat epoxy, which can be attributed to improved dispersion and the formation of interfacial regions that contribute positively to the polarization process. These interfacial regions act as dipole-rich zones, allowing for additional alignment with the applied electric field and thereby increasing the net dielectric storage capability^4^.

At 5 wt.%, a noticeable decline in permittivity is observed. This drop is primarily associated with the formation of immobilized nanolayers, which are rigid polymer regions surrounding the LDH particles. These layers suppress the mobility of polymer chains and inhibit the orientation of nearby dipoles, effectively reducing the material’s ability to store electric energy. The presence of such immobilized zones marks the onset of interfacial rigidity, which counteracts the benefits of polarization introduced at lower concentrations^4,7^. Similar interfacial immobilization effects have been reported in LDH/polymer nanocomposites, where reduced segmental dynamics near LDH layers lead to lower dielectric response^44^.

**Fig. 14:**
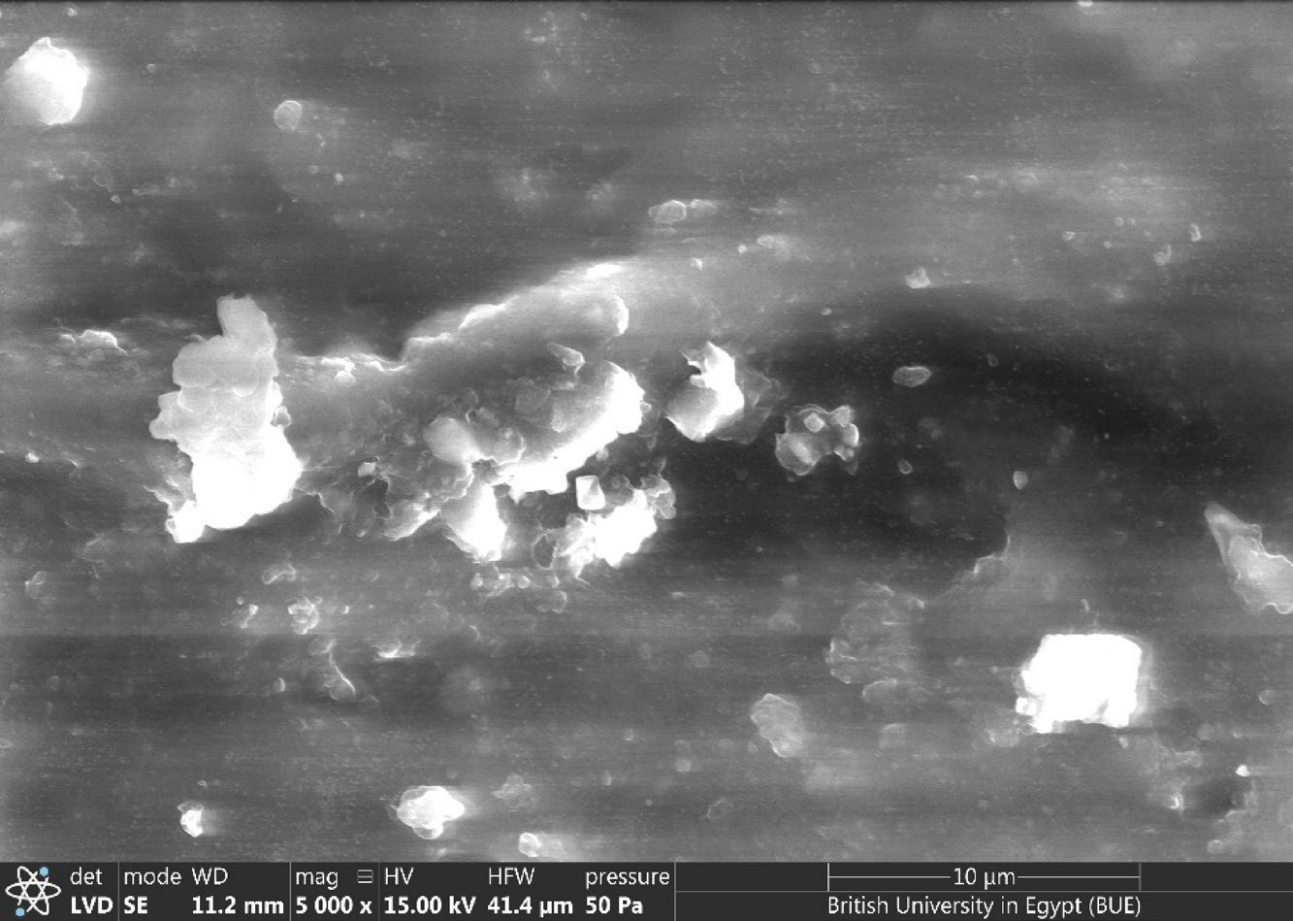
SEM micrograph for 7% Zn/Al-LDH epoxy sample.

At 7 wt.%, the permittivity undergoes a significant reduction, becoming markedly lower than that of the neat epoxy. This decline is indicative of extensive filler agglomeration and the dominance of immobilized nanolayer structures. To provides a more detailed qualitative and quantitative description of the agglomeration behavior at 7 wt.%, a SEM micrograph was conducted as shown in Fig. 14 clearly reveals the presence of dense agglomerates with irregular morphology and poor interparticle dispersion. The observed clusters exhibit sizes ranging from approximately 1 to 6 µm, occupying nearly 30% of the field of view (Fig. 14).

The observed agglomeration reduces the effective interfacial surface area, leading to non-uniform filler distribution and dielectric inhomogeneity. Furthermore, the high filler content amplifies the extent and continuity of the immobilized regions, thereby restricting the dipolar response to larger volumes of the matrix. Consequently, the dielectric contribution from both the matrix and the filler becomes severely diminished^7^.

The intrinsic properties of the LDH structure, specifically those of Zn/Al-LDH, play a central role in shaping this dielectric behavior. The lamellar and ionic nature of LDH particles make them inherently polar, favoring interfacial polarization at moderate concentrations. However, their hydrophilic surfaces and tendency to form hydrogen bonds with the polymer matrix promote the formation of tightly bound immobilized layers. This unique interfacial chemistry, while initially beneficial for polarization, becomes detrimental at higher loadings due to excessive constraint of molecular mobility. Unlike spherical fillers, LDHs introduce anisotropic field distributions and stronger electrostatic interactions at the interface. These characteristics enhance dipole immobilization and facilitate the rapid development of rigid interfacial zones, especially as particle proximity increases^9^.

#### Tan(δ)

**Fig. 15:**
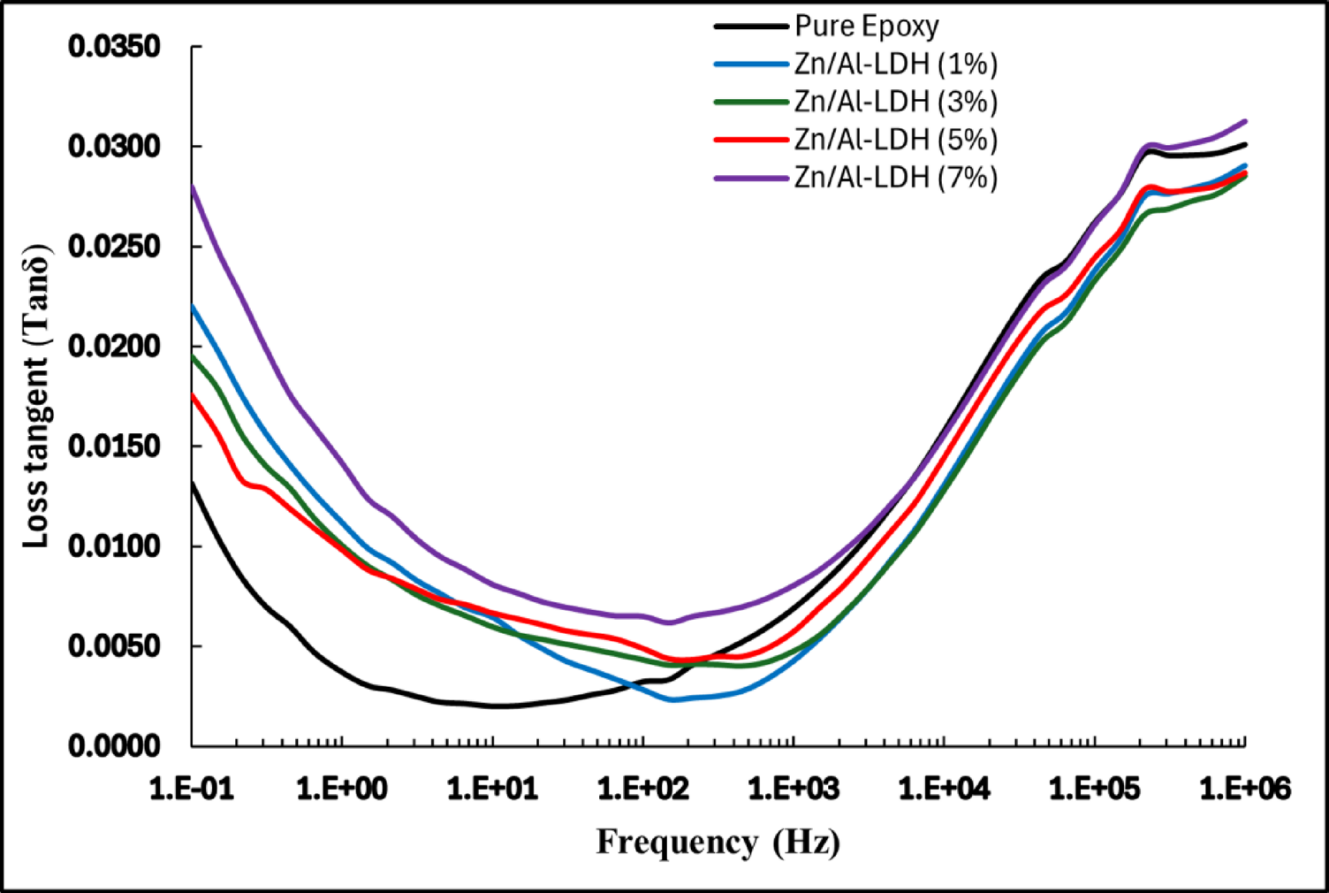
Variation of tan delta with frequency.

The dielectric loss tangent, tan(δ), is a critical parameter that quantifies the phase lag between electric field and polarization response, effectively measuring the proportion of energy dissipated as heat in a dielectric material. For epoxy nanocomposites containing various loadings of Zn/Al layered double hydroxide (LDH), the frequency-dependent behavior of tan(δ) reveals essential information regarding dielectric relaxation, interfacial phenomena, and the balance between permittivity and energy loss. Across the frequency range, as illustrated in Fig. 15, all samples demonstrate a U-shaped tan(δ) curve, with higher values at both low and high frequency extremes and a distinct minimum in the intermediate range (approximately 10–10^3^ Hz).

At very low frequencies (below 1 Hz), dielectric materials, especially polymer nanocomposites, exhibit a significant increase in dielectric loss (tan δ). This behavior is primarily attributed to three mechanisms: conduction loss due to the drift of free or impurity charges under the slow-varying field; interfacial polarization (Maxwell–Wagner–Sillars effect) arising from permittivity and conductivity mismatch between filler and matrix, which leads to charge accumulation at interfaces; and space charge relaxation, where deeply trapped charges gradually respond to the applied field. These combined effects result in higher energy dissipation and elevated loss factors in the low-frequency regime^45^.

This characteristic behavior reflects the combined effects of interfacial polarization at low frequencies and the losses due to immobilized layers at higher frequencies. The intermediate region corresponds to frequencies where polarization mechanisms are most effectively aligned with the applied field, leading to lower loss tangent values.

Adding the Zn/Al-LDH nanofiller with all the weights increases the value of tan(δ) in the region of low frequency below 200 Hz, where the interfacial polarization is dominant. Additionally, it is noticeable that the rate at which the composite eliminates this effect increases as the weight of the nanofiller decreases. On the other hand, adding the nanofillers shows better performance in the high-frequency region, where 1, 3 and 5 wt.% show lower losses than that of the pure epoxy^4^.

The addition of a vertical reference at 50 Hz, corresponding to the standard power frequency in AC electrical systems, provides practical context to the dielectric loss analysis. The tan(δ) values at this frequency, extracted for each filler concentration, show only minor variations across all samples. While slight increases are observed with higher Zn/Al content, particularly at 5 wt.% and 7 wt.%, the changes are modest and well within acceptable design tolerances for high-voltage insulation materials.

#### Conductivity

Electrical conductivity is a critical parameter in assessing the insulation capability of polymer-based materials. For high-voltage applications, particularly under alternating fields or fast switching conditions, the material must exhibit very low conductivity over a broad frequency range to minimize energy losses and prevent current leakage. Frequency-dependent conductivity analysis is thus a powerful diagnostic tool to evaluate the dielectric integrity of epoxy-based nanocomposites subjected to such operational stresses.

The AC conductivity can be extracted from the imaginary part of the complex permittivity using the Eq. (1):

1 $$\upsigma^{\prime} \left( \omega \right) = \omega \varepsilon_{0} \varepsilon ^{\prime \prime} \left( \omega \right)$$

where: σ′ is the real part of conductivity [S/cm], ε₀ is the vacuum permittivity (8.854 × 10⁻^12^ F/m), ε″ is the imaginary part of permittivity, and ω = 2πf is the angular frequency.

**Fig. 16:**
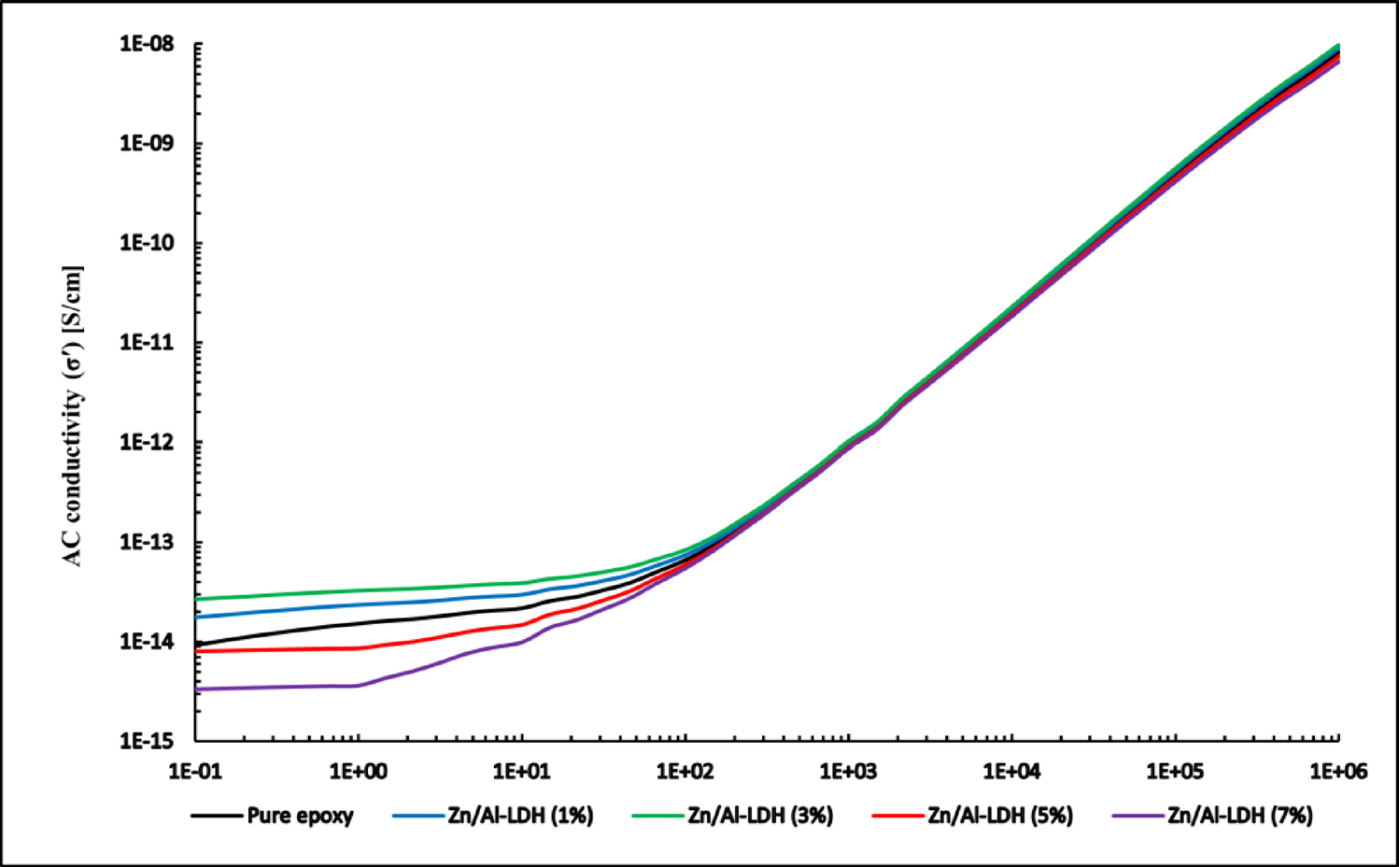
Variation of conductivity with frequency.

Figure 16 illustrates the frequency dependence of the real part of AC conductivity (σ′) for the pure epoxy and Zn/Al–LDH epoxy nanocomposites at various filler loadings (1–7 wt.%), plotted on a log–log scale.

In the low-frequency region approximately up to 100 Hz, the conductivity remains nearly constant, displaying a flat plateau. This behavior corresponds to the dominance of DC conductivity ${(\sigma }_{DC})$, which in this case is extremely low, ranging from 10^−15^ to 10^−13^ S/cm. Such minimal conductivity ensures that the composite behaves as a perfect insulator under static or slowly varying fields, a highly desirable trait for HV insulation systems^4^

Comparing the different loadings, the DC conductivity at low and intermediate frequencies slightly increases up to 3 wt.%, attributed to the formation of additional charge-transfer paths and enhanced interfacial polarization. Beyond this concentration, σ′ decreases, particularly for the 7 wt.% sample, which exhibits the lowest conductivity across the whole frequency range. This reduction is linked to agglomeration of LDH nanorods, which disrupts the homogeneity of the matrix and restricts carrier motion.

In the mid and high frequency region an onset of dispersion occurs at this region marks the transition from frequency-independent to frequency-dependent behavior. σ′ shows a progressive rise for all samples, consistent with Jonscher’s universal power law $\sigma {\prime}(\omega )={\sigma }_{DC}+A{\omega }^{n}$. This behavior indicates that conduction is mainly governed by hopping or tunneling of charge carriers between localized states within the polymer–filler network^4^.

Importantly, even in this regime, the conductivity remains within a safe window (typically below 10^−8^ S/cm), maintaining excellent insulation behavior.

The overall conductivity profile strongly supports the classification of the tested epoxy nanocomposites as high-performance insulating materials. The minimal σ_DC which covers the operating 50Hz frequency in GIS systems, and safe high-frequency behavior corresponding to overvoltage frequencies, all contribute to the material’s reliability in high-voltage environments. These findings are highly encouraging for deployment in GIS, GITL, and other advanced insulation systems requiring stability across wide frequency ranges.

### Breakdown strength performance of epoxy nanocomposites

**Fig. 17:**
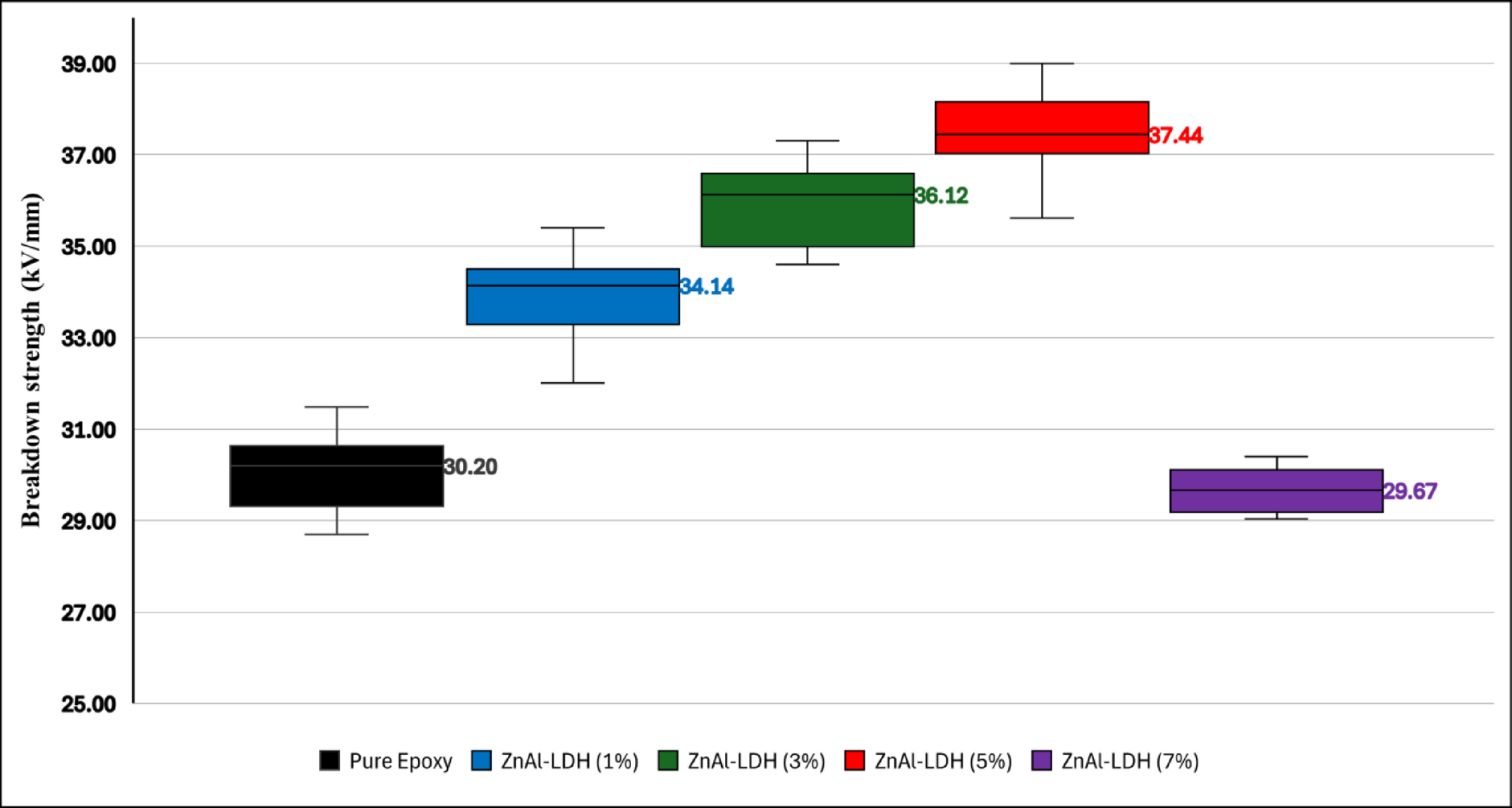
A boxplot visualizes the distribution and identifies outliers in the BDS test.

Epoxy specimens were evaluated in their unfilled (pure) state as well as with Zn/Al-LDH nanofillers at loadings of 1 wt.%, 3 wt.%, 5 wt.%, and 7 wt.%. For each formulation, 15 individual samples were tested. The boxplot shown in Fig. 17 visualizes the distribution of breakdown strength values and clearly indicates the median. Outliers were identified according to Tukey’s 1.5 × IQR criterion, where the interquartile range (IQR) represents the difference between the third (Q3) and first (Q1) quartiles. According to this criterion, no measured value was found to be an outlier.

The statistical summary in Table 3 reveals a nonlinear enhancement in BDS with increasing Zn/Al concentration. Specifically, the neat epoxy exhibited a mean value of 30.08 kV/mm with a standard deviation of 0.91. The incorporation of 1 wt.% Zn/Al increased the mean BDS to 33.90 kV/mm, and the addition of 3 wt.% Zn/Al further improved to 36.06 kV/mm. The maximum BDS was observed at 5 wt.%, reaching 37.42 kV/mm, beyond which a substantial decline occurred at 7 wt.%, falling back to 29.68 kV/mm, which is even lower than that of the neat epoxy. This trend suggests that an optimum nanofiller loading of between 3 and 5% wt. maximizes dielectric strength.

**Table 3:** Basic statistics for the BDS test.

Material	Min (kV/mm)	Max (kV/mm)	Mean (kV/mm)	Std Deviation (kV/mm)	Coefficient of Variation (%)
Pure Epoxy	28.7	31.5	30.08	0.91	2.03
Zn/Al-LDH (1%)	32	35.4	33.9	0.88	2.61
Zn/Al-LDH (3%)	34.6	37.3	36.06	0.9	2.5
Zn/Al-LDH (5%)	35.6	39	37.42	0.99	2.64
Zn/Al-LDH (7%)	29	30.4	29.68	0.49	3.66

The coefficient of variation (CV) supports the same conclusion. The 5 wt.% sample not only had the highest BDS mean but also maintained a low CV of 2.64%, indicating highly stable and reproducible breakdown behavior. The observed behavior can be rationalized in terms of interfacial polarization, filler dispersion quality, and immobilized interfacial regions, consistent with the permittivity data. At low filler concentrations (1–3 wt.%), the improved BDS is attributed to the formation of well-dispersed interfacial zones between the Zn/Al-LDH particles and the polymer matrix. These regions enhanced dielectric polarization, suppressed local electric field intensities, and introduced charge trapping sites at the polymer filler interfaces, which capture injected charges and disrupt the continuous streamer channels responsible for breakdown propagation.

This interpretation is supported by the slight increase in ε′ at 3 wt.% and the moderate tan(δ) values, indicating efficient energy storage with minimal dielectric loss.

At 5 wt.% Zn/Al, the composite appears to reach an optimal balance. The permittivity analysis confirms that while ε′ slightly decreases, the slight suppression in ε″ and tan(δ) at this concentration suggests that the immobilized nanolayers formed around the fillers act as deep charge traps, stabilizing the internal field distribution and boosting breakdown resistance.

Moreover, the inherent nature of Zn/Al as a layered double hydroxide (LDH) further amplifies these effects. The lamellar structure of LDHs provides a high interfacial area, facilitating strong hydrogen bonding with the epoxy matrix and promoting the formation of immobilized interfacial nanolayers. These features not only enhance interfacial polarization at moderate concentrations but also increase the interfacial trap density and charge retention capacity^9^.

Interfacial polarization facilitates charge trapping and mitigating local electric stress by redistributing the electric field and creating localized regions of charge accumulation at the filler matrix interface. At optimized filler loadings, this reduces field concentration and delays breakdown. However, excessive interfacial polarization due to agglomeration or high filler content can instead lead to localized field intensification.

**Fig. 18:**
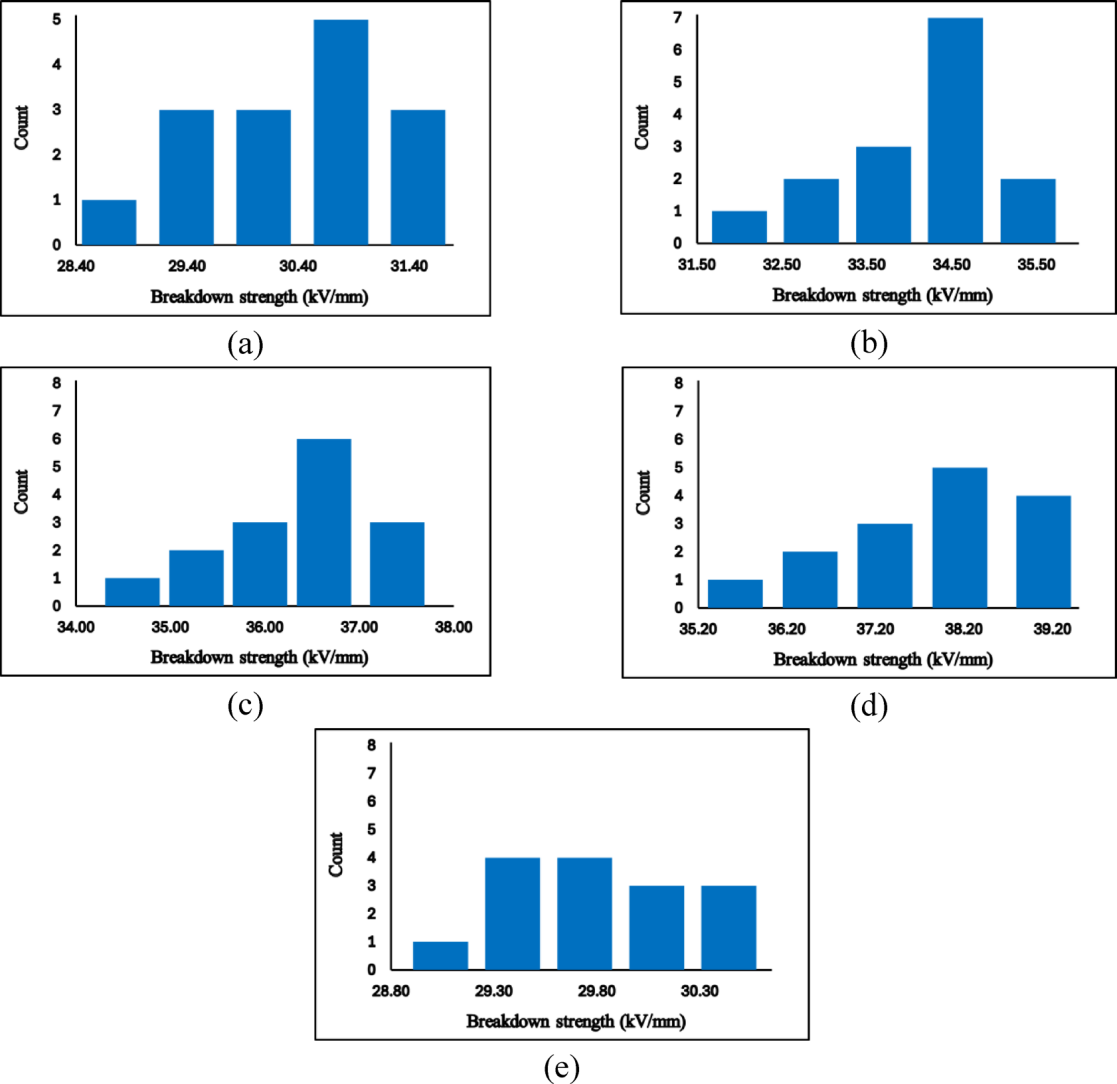
A histogram shows the frequency distribution of BDS values. (**a**) pure epoxy, (b) Zn/Al (1%), (**c**) Zn/Al (3%), (d) Zn/AL (5%), and (e) Zn/Al (7%).

The histogram plots presented in Fig. 18 provide a clear statistical visualization of the breakdown strength (BDS) measurements for all epoxy and Zn/Al–LDH nanocomposite formulations. These distributions offer critical insights into the repeatability, stability, and intrinsic variability of the experimental data, thereby supporting the validity of the subsequent Weibull reliability analysis.

Across all samples, the histograms exhibit well-defined, unimodal distributions, indicating that the BDS values cluster around a dominant central tendency with limited scattering. This behaviour reflects a high degree of measurement consistency and suggests that the specimens within each formulation respond in a statistically uniform manner. The frequency profiles also reveal the absence of extreme outliers or irregular secondary peaks, demonstrating that the fabrication process yielded samples with reproducible breakdown behaviour. Such well-behaved distributions are essential in high-voltage dielectric studies, as they confirm that the observed improvements are intrinsic to the material system rather than artefacts of random experimental fluctuation.

Furthermore, the compactness of the histograms for the optimal filler loadings implies a reduction in stochastic variability typically associated with microstructural defects and localized weak points. This observation reinforces the hypothesis that the incorporation of Zn/Al–LDH at moderate concentrations promotes a more uniform dispersion and contributes to a more robust dielectric network capable of sustaining higher electric fields before failure.

#### Weibull distribution

Weibull analysis plays a crucial role in interpreting dielectric breakdown strength (BDS) data, particularly for polymeric insulation materials. It offers a deeper understanding of the distribution of breakdown events: the shape parameter (β) reflects the scatter of data, indicating the uniformity and consistency of failure. In contrast, the scale parameter (α) provides a measure of the characteristic breakdown strength at which 63.2% of the specimens are expected to fail.

The two-parameter Weibull cumulative distribution function is given by the Eq. (2):

2 $$f_{\left( x \right)} = 1 - \exp \left[ { - \left( {\frac{x}{{\upalpha }}} \right)^{{\upbeta }} } \right]$$

where: F(x) is the cumulative probability of failure at stress level x, α is the scale parameter which describes the breakdown strength at which 63.2% of samples are expected to fail, and β is the shape parameter which a dimensionless parameter indicating the dispersion and failure mode.

To linearize the equation and enable graphical interpretation, the double logarithmic form is often used:

3 $$\ln \left[ { - \ln \left( {1 - f_{\left( x \right)} } \right)} \right] = \beta \ln \left( x \right) - \beta \ln \left( \alpha \right)$$

4 $${\mathrm{y}} = {\upbeta } \ln \left( {\mathrm{x}} \right) - {\upbeta }\ln \left( {\upalpha } \right)$$

This transformation allows for a straight-line fit on a plot of ln(x) versus ln [-ln (1–F)], where the slope represents β and the intercept is related to − βln(α). To apply the two-parameter Weibull distribution to the breakdown strength data, the measured values are first arranged in ascending order. The cumulative probability of failure for each ranked value is then estimated using Benard’s approximation formula (5), then by plotting the double logarithmic form (3), a straight line is obtained whose slope represents the shape parameter β, while the scale parameter α is derived from the intercept $-\beta In\left(\alpha \right)$ as shown in Eq. (4):

5 $${\mathrm{f}}\left( {{\mathrm{x}}_{{\mathrm{i}}} } \right) = \frac{{{\mathrm{i}} - 0.3}}{{{\mathrm{n}} + 0.4}}$$

where: i is the rank of the value x_i_ from 1 to n, and n is the total number of data points.

To assess how well the Weibull curve fits the actual data points, the coefficient of determination R^2^ is calculated for each data set. The R^2^ values obtained for all samples fall within or above the generally accepted threshold for good statistical fit (≥ 0.90), confirming the suitability of the Weibull model in representing the breakdown behavior. All formulations exhibited strong linearity except for the 7 wt.% Zn/Al-LDH sample, which showed a noticeably lower R^2^ value (0.877), likely due to nanofiller agglomeration and increased variability in dielectric performance.

Additionally, the summary of the Weibull parameters in Table 4 indicates that the addition of Zn/Al nanofillers yields a notable enhancement in dielectric performance, particularly at a 5 wt% concentration. The scale parameter α increased significantly from its baseline value in pure epoxy, indicating that most breakdown events in the nanocomposite occurred at higher electric field levels. This is consistent with the experimental observation of an improvement in dielectric strength. Similarly, the shape parameter β increased with the addition of 1%, 3%, and 5% Zn/Al, reflecting reduced variability and more homogeneous failure behavior, likely due to better field distribution and interface interaction.

**Table 4:** Weibull parameters of all data sets.

Material	R^2^	α (kV/mm)	β
Pure Epoxy	0.9292	30.5	39.23
Zn/Al-LDH (1%)	0.972	34.31	46.26
Zn/Al-LDH (3%)	0.92	36.48	47.34
Zn/Al-LDH (5%)	0.9632	37.87	45.75
Zn/Al-LDH (7%)	0.877	29.91	69.77

However, at 7 wt.%, α is declined, signaling a deterioration in insulation quality. This behavior is attributed to the possible agglomeration of excess nanofiller particles, which may introduce local field enhancements and micro voids, acting as premature breakdown sites. This explanation is supported by the earlier permittivity analysis, which showed signs of interfacial polarization saturation and increased dielectric loss at higher concentrations, indicating a limit to the beneficial role of filler loading.

The high shape parameter value β should not be interpreted as improved reliability or superior insulation performance. Instead, it reflects the fact that, for the 7 wt.% composite, almost all specimens fail within a very narrow low-field range (29–30.4 kV/mm), as also seen from the basic statistics in Table 3 and the histogram in Fig. 18.

**Fig. 19:**
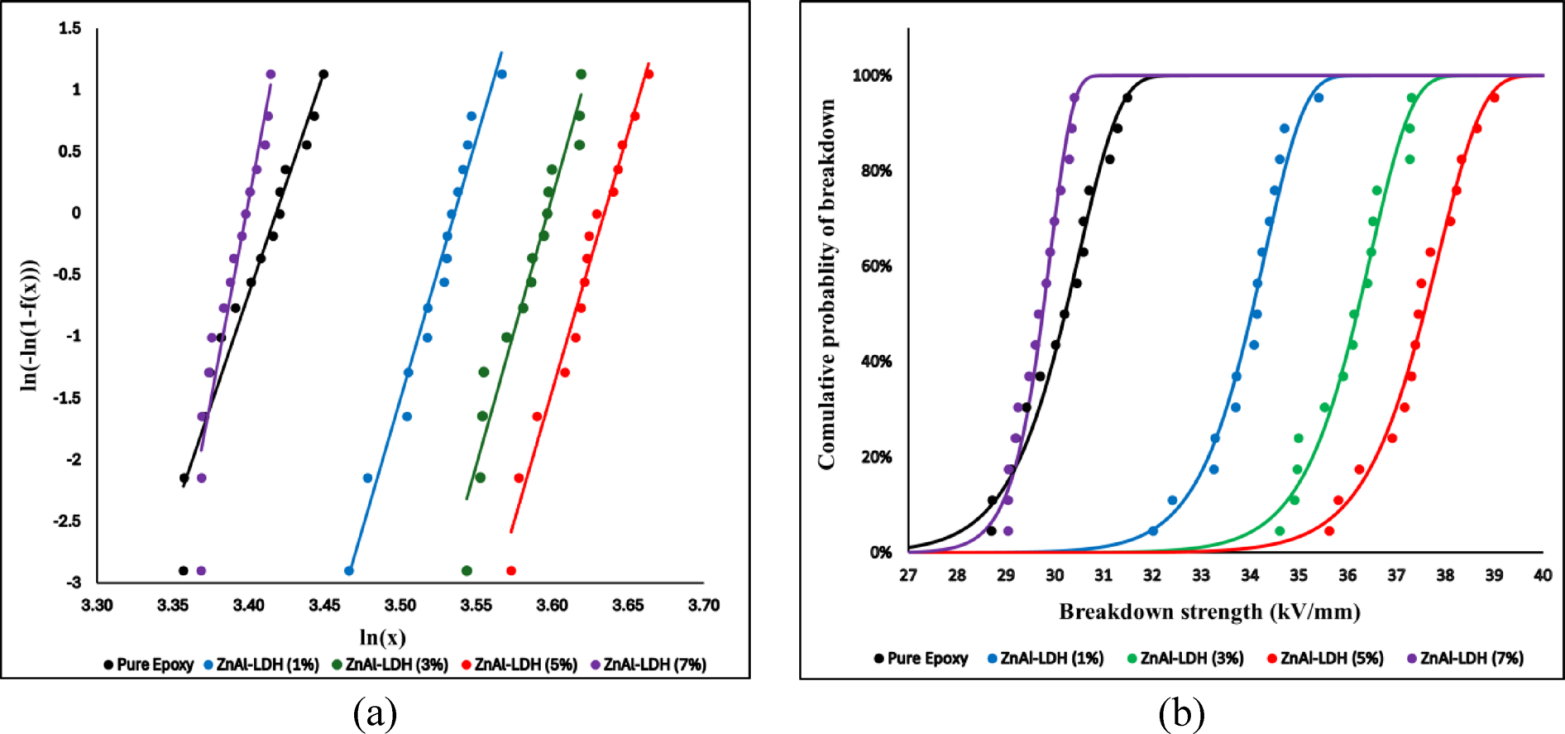
The logarithmic and linear scale Weibull plot of all material samples.

The logarithmic Weibull plot depicted in Fig. 19, confirms the model’s applicability, showing a clear linear relationship across all formulations. The steepest slope and furthest rightward shift were observed at 5 wt.%, corroborating the superior performance and reliability at this loading. Meanwhile, the cumulative probability plot illustrated the distribution of breakdown events, and the horizontal α-line (F = 63.2%) allowed direct comparison of characteristic strength across all samples. The gradual shift of the α-intercept toward higher voltages, up to 5 wt.%, and its retreat at 7 wt.%, visually support the statistical trends.

Another fundamental aspect that can be obtained from Weibull analysis is the reliability curve, which can be used in evaluating the performance and consistency of insulating materials under high-voltage stress. While traditional metrics such as the mean breakdown strength provide insight into the average performance, they fail to capture the statistical probability of failure at specific stress levels. This is where Weibull-based reliability assessment becomes essential, as it offers a more robust evaluation by considering both the strength distribution and the associated failure probabilities.

The equation defines the reliability function R(x) for a Weibull-distributed variable (**6**):

6 $${\mathrm{R}}_{{\left( {\mathrm{x}} \right)}} = \exp \left[ { - \left( {\frac{{\mathrm{x}}}{{\upbeta }}} \right)^{{\upalpha }} } \right]$$

where: ${\mathrm{R}}_{\left(\mathrm{x}\right)}$ is the reliability at stress x.

**Fig. 20:**
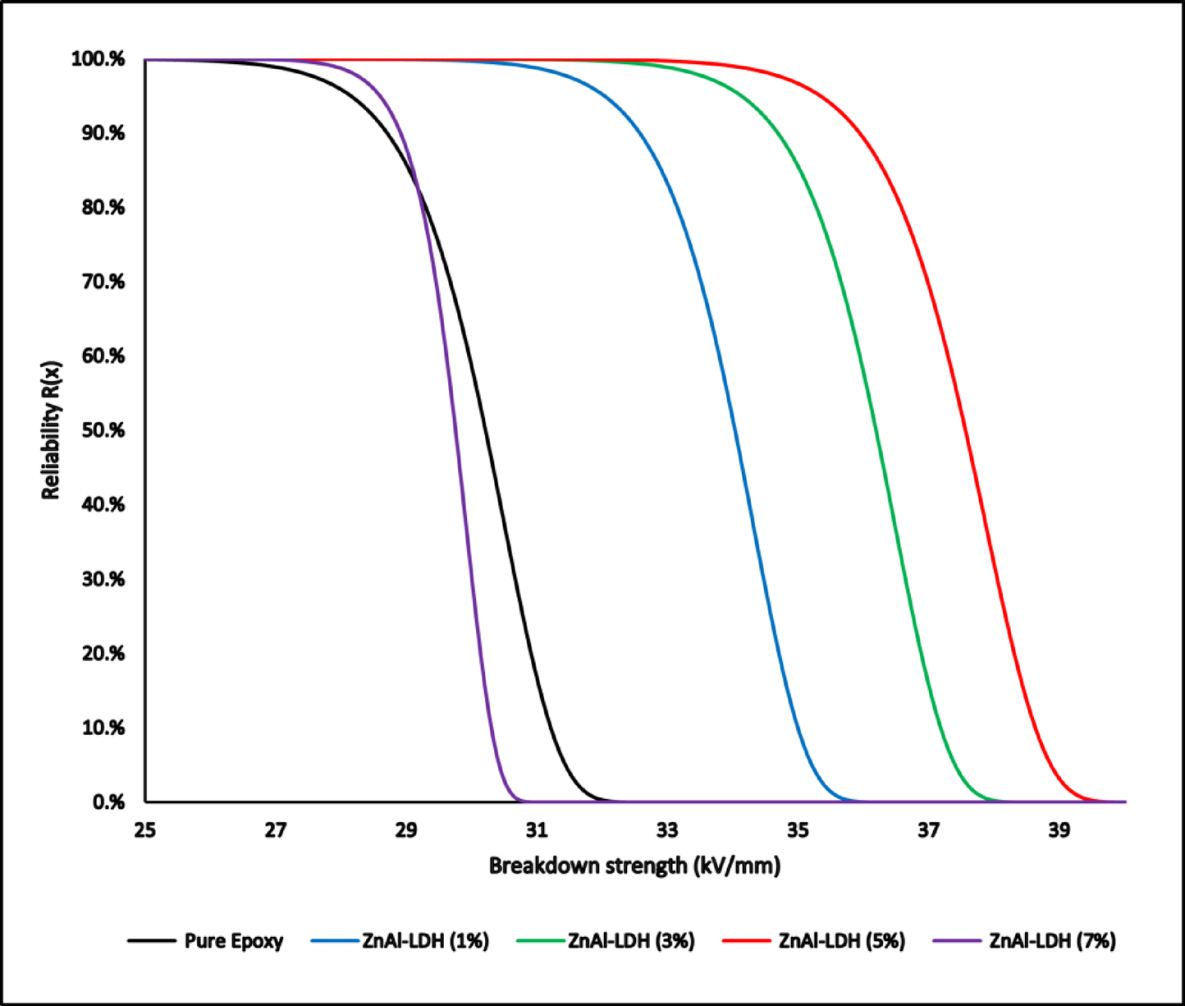
The Weibull reliability curves of all materials.

The Weibull reliability curves are presented in Fig. 20 For all tested compositions. These curves represent the probability that each sample will withstand a given stress without breaking down. The results clearly show that adding Zn/Al-LDH nanofillers enhances reliability across the breakdown strength range. The 3 wt.% and 5 wt.% samples demonstrated the most favorable profiles, shifting the curves toward higher field values and indicating improved dielectric performance. In contrast, the 7 wt.% sample exhibited reduced reliability, likely due to nanoparticle agglomeration and interfacial degradation, which introduced local defects and field concentration points.

## Conclusion

This work investigated the potential of Zn/Al layered double hydroxide (LDH) nanorods as functional fillers to enhance the dielectric and thermal performance of epoxy resin, aiming at their utilization in Gas-Insulated Switchgear (GIS) and Transmission Line (GITL) spacer applications.

A comprehensive experimental approach was adopted: the LDH nanofillers were synthesized, structurally characterized, and incorporated into epoxy at different loadings (1, 3, 5, and 7 wt.%). The resulting nanocomposites were then systematically evaluated through dielectric spectroscopy, breakdown strength testing, thermal analysis, and microstructural characterization. The key findings can be summarized as follows:

1. Zn/Al-LDH nanoparticles exhibited a nanocrystalline, semi-ordered layered structure with surface area ~ 98.9 m^2^/g. Also, the mesoporous architecture with an average pore diameter of ~ 18.7 nm provided high adsorption capacity and effective charge-trapping capability, both advantageous for electrical insulation.
2. The SEM/EDX validated the presence of Zn and Al uniformly distributed in the polymeric background with confirmation of the successful incorporation of LDH into the epoxy matrix, increasing structural ordering from 21.1% for neat epoxy to 28.9% for 5 wt.% composite.
3. TGA showed improved thermal stability: char residue increased from 14% for neat epoxy to 17.1% for 5 wt.% composite. DSC revealed an increase in glass transition temperature from 93.5 °C to 109.8 °C at 5 wt.%, surpassing the IEC 62271-1 operational requirement of 105 °C.
4. Relative permittivity (ε′) improved slightly at 1–3 wt.% but declined at higher loadings (5–7 wt.%) due to interfacial constraints and agglomeration. Dielectric loss (tan δ) stayed within safe limits at 50 Hz, and AC conductivity was suppressed to 10^−8^ S/cm, confirming strong insulating performance
5. The breakdown strength improved steadily with fillers loading up to 5 wt.%, reaching 37.4 kV/mm, which is ≈24% higher than that of neat epoxy. At this level, the composite also showed the most consistent performance. Beyond 5 wt%, however, agglomeration effects dominated, and the strength dropped below that of pure epoxy. Thus, the optimum loading is clearly at 5 wt.%.

Although the results confirm the significant potential of Zn/Al-LDH/epoxy nanocomposites for high-voltage insulation applications, this study was limited to short-term laboratory conditions. Further research is needed to evaluate the long-term aging stability, humidity influence, and electrical endurance under service-like stress environments. Such investigations would provide deeper insight into the reliability and field applicability of LDH-based epoxy materials in GIS/GITL systems.

## Data Availability

Data is available with all authors and will be made accessible to others upon reasonable request.
